# Dynamics of Internalization and Intracellular Interaction of Tau Antibodies and Human Pathological Tau Protein in a Human Neuron-Like Model

**DOI:** 10.3389/fneur.2020.602292

**Published:** 2020-11-26

**Authors:** Dov B. Shamir, Yan Deng, Qian Wu, Swananda Modak, Erin E. Congdon, Einar M. Sigurdsson

**Affiliations:** ^1^Department of Neuroscience and Physiology, Neuroscience Institute, New York University Grossman School of Medicine, New York, NY, United States; ^2^Microscopy Core, New York University Grossman School of Medicine, New York, NY, United States; ^3^Department of Psychiatry, New York University Grossman School of Medicine, New York, NY, United States

**Keywords:** Alzheimer's disease, tau, PHF, antibodies, immunotherapies, live imaging

## Abstract

We and others have shown in various *in vivo, ex vivo* and cell culture models that several tau antibodies interact with pathological tau within neurons. To further clarify this interaction in a dynamic human model, we differentiated SH-SY5Y cells with retinoic acid and BDNF to create a neuron-like model. Therein, tau antibodies were primarily taken up by receptor-mediated endocytosis, and prevented toxicity of human brain-derived paired helical filament-enriched tau (PHF). Subsequently, we monitored in real-time the interaction of antibodies and PHF within endocytic cellular compartments. Cells were pre-treated with fluorescently-tagged PHF and then incubated with tau antibodies, 4E6, 6B2, or non-specific isotype control IgG1 labeled with a pH sensitive dye. The uptake and binding of the efficacious antibody, 4E6, to PHF occurred mainly within the soma, whereas the ineffective antibody, 6B2, and ineffective control IgG1, were visualized via the processes and showed limited colocalization with PHF within this period. In summary, we have developed a neuron-like model that clarifies the early intracellular dynamics of the interaction of tau antibodies with pathological tau, and identifies features associated with efficacy. Since the model is entirely human, it is suitable to verify the therapeutic potential of humanized antibodies prior to extensive clinical trials.

## Introduction

Immunotherapies targeting pathogenic peptide/protein aggregates are at different stages of development as potential treatment for Alzheimer's disease (AD) and other neurodegenerative diseases (1–4). Our laboratory pioneered targeting pathological tau protein with active and passive immunotherapies for AD and other tauopathies (5, 6), which have been validated and extended by other groups over the last several years and now entered clinical trials (1–4). Antibody-mediated clearance of tau likely involves several mechanisms, which may include: (a) microglia activation and phagocytosis (7–10), (b) neutralization of tau in the extracellular space (11, 12), and (c) intracellular sequestration/degradation of tau within neurons (5, 7, 13–24). Our group and others have focused on elucidating these mechanisms in various cell culture, *ex-vivo*, and *in-vivo* mouse models. To validate humanized antibodies for clinical trials, models of human origin should ideally be employed, and live imaging at the early stages of treatment may provide valuable insight into the mechanisms involved that cannot be obtained by other means. Not much is known about the interaction and internalization of pathological tau and its antibodies in the first hours of treatment.

We previously showed that colocalization of tau with antibodies is primarily within the soma of neurons using *in vivo, ex vivo* and culture models of mouse origin (5, 7, 12, 14, 16, 22, 23). Regarding the specific antibodies examined herein, 4E6 and 6B2, we have previously shown that both are readily taken up into tauopathy mouse neurons in brain slices and primary culture models, where they colocalize with tau primarily in the endosomal lysosomal system, and clear soluble tau, with 4E6 more consistently being effective in clearing pathological tau and preventing its toxicity (7, 12, 14, 23). However, only 4E6 and not 6B2 is effective in clearing tau pathology *in vivo* (12, 22), which is associated with cognitive improvements (12). In addition, we have previously shown extensive internalization of the 6B2 antibody and pathological tau derived from human brain in non-differentiated neuroblastoma model using flow cytometry (19).

In the prior mouse studies, we showed that both the effective 4E6 tau antibody and the relatively ineffective 6B2 tau antibody could localize with somatic intraneuronal tau in mouse brain slices and in mouse primary neurons following an incubation for 24 h or longer. Those findings do not provide insight into why the former is effective and the latter ineffective. Therefore, we set out to determine with live imaging if earlier cellular events, like temporal and spatial differences, might provide better insight into this important issue as both antibodies are clearly taken up into the endosomal-lysosomal system. For early high throughput screening, differentiated neuroblastoma cells are preferred over primary neurons for live imaging as they are less sensitive to the conditions for such analysis. Therefore, we established a neuron-like tauopathy model of human origin that shows the expected neuro- and synaptotoxicity of human-derived paired helical filament (PHF)-enriched pathological tau, and the prevention of its deleterious effect by a tau antibody. Importantly, naïve and differentiated cells utilize different mechanisms of antibody uptake and the tau antibody is only effective in the differentiated more neuron-like cells. Furthermore, live imaging revealed that in differentiated neurons pre-treated with PHF-tau, 4E6 tau antibody, which prevents PHF-tau toxicity, rapidly colocalized with PHF-tau within the soma. In contrast, 6B2 tau antibody and control IgG1, which do not prevent PHF-tau toxicity, were taken up via the neuronal processes and did not colocalize with PHF-tau within this timeframe. This model and experimental design should provide valuable insight into the efficacy and mechanisms of action of tau antibodies that are candidates for clinical trials.

## Materials and Methods

### Paired Helical Filament (PHF) Protein Preparation

Human brain slices from subjects with extensive amyloid-β plaques and neurofibrillary tangles were enriched for PHF for the experiments. Brain slabs were homogenized and prepared as described by us and others (12, 18, 19, 25, 26), and the enriched PHF had similar properties with regard to western profile, ratio of 3R/4R tau and toxicity in culture. Briefly, the brain tissue was homogenized and prepared in buffer [pH 6.5; 0.75 M NaCl, 1 mM EGTA, 0.5 mM MgSO_4_, and 100 mM 2-(N-morpholino) ethanesulfonic acid] along with protease inhibitor cocktail (Roche) and centrifuged at 11,000 x g for 20 min at 4°C. Supernatant was subsequently centrifuged in an ultracentrifuge at 100,000 x g for 60 min at 4°C. The pellet was then resuspended in paired helical filament (PHF) extraction buffer containing sucrose (10 mM Tris; 10% sucrose; 0.85 M NaCl; and 1 mM EGTA, pH 7.4) and spun at 15,000 x g for 20 min at 4°C. The pellet was extracted again in the sucrose buffer at the same low-speed centrifugation. The supernatants from both sucrose extractions were pooled and subjected to 1% sarkosyl solubilization by briefly heating it and then stirring at ambient temperature, and then centrifuged at 100,000 x g for 60 min at 4°C in a Beckman 60 Ti rotor (Beckman Coulter; Fullerton, CA). The resulting pellet was re-suspended in 50 mM Tris-HCl (pH 7.4), using 0.5 mL of buffer for each milligram of initial weight of brain sample protein. It was then dialyzed in PBS overnight at 4°C, using a 3,500 MW cassette (Thermo Scientific), and designated the PHF enriched preparation.

### Antibodies and Fluorescence Labeling

In this study we used tau antibodies, 6B2 and 4E6, that have been generated against the Tau386-408[P-Ser396, 404] region. These IgG1κ mouse antibodies were previously characterized by our laboratory (7, 12, 14, 16, 19, 22, 23, 27). As a control, a non-specific mouse IgG1κ (IgG1, eBioscience) antibody was used. The antibodies were tagged with Cypher5E (GE Healthcare) fluorescent marker where mentioned. Cypher5E is a pH sensitive dye, and only fluoresces within acidic compartments, like endosomes or lysosomes. The PHF-enriched brain fraction was tagged with Alexa Fluor 488 (Invitrogen) where mentioned. All tagging was performed as outlined in the manufacturer's instructions.

### Cell Culture and Differentiation

SH-SY5Y human neuroblastoma cells were obtained from ATCC. Cells were cultured in complete media (Dulbecco's Modified Eagle Medium (DMEM) with GlutaMAX (Invitrogen), 10% heat inactivated fetal bovine serum (FBS), 10,000 Units/mL Penicillin, and 10,000 μg/mL streptomycin. Cells were plated at 4 × 10^2^ cells/mm^2^, allowed to recover for 3 to 5 days before each experiment, and grown in an incubator with 5% CO_2_ at 37°C.

Cells were maintained, prior to differentiation or treatment, in complete media. The cells were double-differentiated as described by other groups (28–30), by adding 10 μM retinoic acid (RA, Sigma Aldrich) and 1% FBS for 5 days. Then after two washes in DMEM media, they were treated with 50 ng/mL brain-derived neurotrophic factor (BDNF, Alomone Labs) for at least 2 days. For all experiments, the differentiated cells (DC) were then treated with the BDNF media, while non-differentiated cells (NDC) were grown and treated in DMEM media with 1% fetal bovine serum (FBS). For characterization experiments, both cell types were treated with 5 μg/mL 6B2 tau antibody for 24 h, and for clathrin receptor inhibition studies, followed by increasing amounts of inhibitor, Dynasore (0–5 μg/mL). For PHF dose response studies, SH-SY5Y were treated with tagged PHF (0–10 μg/mL) for 24 h, washed, and then incubated for another 24 h. For pre-incubation studies analyzed using Western blot, cells were pre-treated with PHF (1 or 10 μg/mL) for 24 h, then washed with DMEM to remove remaining extracellular PHF, and subsequently incubated with 5 μg/mL tau antibodies (6B2 or 4E6) or IgG1 control for another 24 or 120 h.

### Western Blot

All cell lysates analyzed using Western blot were incubated with non-tagged antibodies (6B2, 4E6, and IgG1) and/or PHF. Prior to cell lysis, cells were thoroughly washed with PBS. In preliminary studies, we verified by confocal imaging using fluorescently labeled antibodies and PHF that this method cleared all extracellular antibodies/PHF. Under our conditions, trypsin digestion had the same effect but did damage the cell membranes to a variable degree, which included some membrane rupture and extracellular release of its contents including antibodies and PHF. Hence, we opted for the milder repeated PBS wash. All samples were then homogenized in RIPA buffer and prepared as described previously (7). Samples were boiled and loaded onto 10% SDS-PAGE gels, electrophoresed, and then transferred to nitrocellulose membranes, which were blocked in 5% milk with 0.1% TBS-T. Blots were then probed for total tau (Dako polyclonal antibody), NeuN (Millipore polyclonal antibody), synaptophysin (Sigma Aldrich monoclonal antibody), PSD-95 (UC Davis NeuroMab, monoclonal antibody), or GAPDH (Abcam polyclonal antibody) primary antibodies overnight at 4°C, washed and then probed with anti-horseradish peroxidase (HRP) conjugated rabbit or mouse secondary antibody (Pierce) for 1 h. For antibody uptake detection, membranes were incubated with an anti-mouse IgG1 HRP-conjugated secondary antibody with specificity against the heavy chain (Bethyl Laboratories), and signal was detected with an ECL substrate (Thermo Scientific). Images of immunoreactive bands were then acquired, normalized relative to internal controls and quantified using the Fuji LAS-4000 imaging system. For immunoprecipitation studies, blots were incubated with anti-CD16 (Santa Cruz, RRID: AB_2104020), anti-CD32 (Santa Cruz, RRID: AB_2103599) polyclonal antibodies overnight at 4°C. All blots were washed and probed with a fluorescent anti-rabbit secondary antibody at a 1:10,000 dilution (LI-COR, RRID:AB_621843), and bands were visualized using a LI-COR Odyssey CLX reader.

### Immunoprecipitation

Neuroblastoma SH-SY5Y cells were maintained and differentiated as described above. Primary neurons were prepared from a wild type pup at postnatal day 0 as previously described (12, 14, 23). All of the samples were homogenized in RIPA buffer (50 mM Tris-HCl (pH 7.4), 150 mM NaCl, 1 mM ethylenediaminetetraacetic acid (EDTA), 1 mM phenylmethylsulfonyl fluoride (PMSF), 1 mM NaF, 1 mM Na_3_VO_4_, 1 μg/ml complete protease inhibitor cocktail (Roche Applied Science) and assayed for protein concentration. Equal amounts of total protein from each sample was then used for immunoprecipitation.

Immunoprecipitation was carried out using a magnetic Dynabead kit (ThermoFisher) per manufacturer's instructions. Briefly, beads were incubated with an antibody recognizing mouse FcγII/III receptors (eBioscience). Beads were then washed and equal amount of protein from each sample was added. Following incubation, the beads were then washed again, and the target protein eluted. O+ buffer [62.5 mM Tris-HCl (pH 6.8), 5% glycerol, 5% β-mercaptoethanol, 2.3% SDS, 1 mM EDTA, 1 mM ethylene glycol bis(2-aminoethyl) tetraacetic acid (EGTA), 1 mM PMSF, 1 mM NaF, 1 mM Na_3_VO_4_ and 1 μg/ml complete protease inhibitor cocktail (Roche Applied Science)] was added and samples were boiled before loading them onto an SDS gel for immunoblotting.

### RNA Extraction and PCR

Cells were washed thoroughly with PBS and then lysed and total RNA extracted using a kit as described by manufacturer's instructions (Sigma). RNA was then converted to cDNA using reverse transcriptase kit according to manufacturer's instructions (Invitrogen). The cDNA samples were then amplified by PCR using the following primers from Sigma; FcγR2A (NM_001136219), FcγR3A (NM_000569), FcγR3B (NM_001244753), and Actin (NM_001101). All amplified samples were run on a 2% agarose gel and their predicted size confirmed by DNA ladders. DNA bands, representing the extracted RNA, were stained with ethidium bromide, imaged by Protein Simple, Alpha-Imager HP system, and analyzed using ImageJ.

### Flow Cytometry

All cells analyzed with flow cytometry were incubated with tagged PHF, and prepared for flow cytometry as previously described (19). Cells were analyzed using FlowJo for Alexa Fluor 647 positive cells. Median fluoresence intensity (MFI) values were obtained for fluorescent signals.

### Immunohistochemistry and Cell Staining

Coverslips were coated with Pluripro protein matrix (Cell Guidance Systems) as directed by the manufacturer. Cells were plated onto coverslips at 4 × 10^2^ cells/mm^2^, and then allowed to recover for 2 days. For characterization studies, NDC and DC were co-incubated with Dextran (10,000 MW, Alexa Fluor 568, Invitrogen) and 20 μg/mL Alexa Fluor 488 tagged 6B2 tau antibody. Prior to fixation, cells were incubated with Hoechst nuclear staining, as described by the manufacturer (Invitrogen), then fixed with 4% paraformaldehyde, and coverslipped.

### Live Cell Imaging

Chamber glasses (Nunc) were coated with Pluripro protein matrix (Cell Guidance Systems) as directed by the manufacturer. Cells were plated onto these glasses at 2 × 10^2^ cells/mm^2^ and allowed to recover for 2 days. Cells were then differentiated as described above. For all pre-incubation studies, cells were then treated with 50 μg/mL fluorescently tagged PHF for 16 h. Prior to live imaging for all experiments, Hoechst nuclear dye was incubated with the live cells as directed by the manufacturer (Invitrogen). Cells were then washed and placed into complete media with no phenol red-DMEM and HEPES (Invitrogen) with BDNF for differentiated cells.

As previously described for time-lapse studies by our group (31), following Hoechst staining, cells were then incubated with tagged 20 μg/mL 4E6, 6B2, IgG1, or 50 μg/mL PHF for 140–150 min, which was added to the chamber glasses within the first 5 min of observation in the live imaging chamber. Cells were imaged every 5 min for up to 140–150 min. Chamber glasses were analyzed using an API DeltaVision PersonalDV system with Olympus PlanApo N 60x/1.42 oil lens, standard fluorescent filterset and full environmental control device. The internal density (IntDen) of either the antibody or PHF signal of the whole image was extracted using ImageJ. In these time-lapse studies, colocalization analysis of the antibody-dextran and time-lapse images were conducted using ImageJ's Intensity Correlation Analysis plugin as previously described by us and others (16, 22, 32–34). Degree of colocalization coefficient (*R*^2^) and product of difference from the mean (PDM) images of corresponding experimental groups were calculated and displayed, where pixels in yellow indicate colocalizing in both channels and blue indicates a negative correlation.

### Statistics

All quantified Western blot, flow cytometry, PCR, or live imaging data was analyzed using GraphPad Prism 6. ANOVA or Student's *t*-test were used to compare results of different samples. For three or more groups, one- or two-way ANOVA was used, depending on the number of variables, followed by Bonferroni *post hoc* test. Student's *t*-test was one or two-tailed as specified, depending on the analyzed parameters. The coefficient of determination (*r*^2^) and trend line were generated for degree of colocalization coefficient (*R*^2^) vs. time data in the live time-lapse imaging studies, which was further analyzed using Pearson correlation coefficient.

## Results

### Differentiation of SH-SY5Y Neuroblastoma Cells Increases Tau Levels and Decreases Antibody Internalization

**Figure 1:**
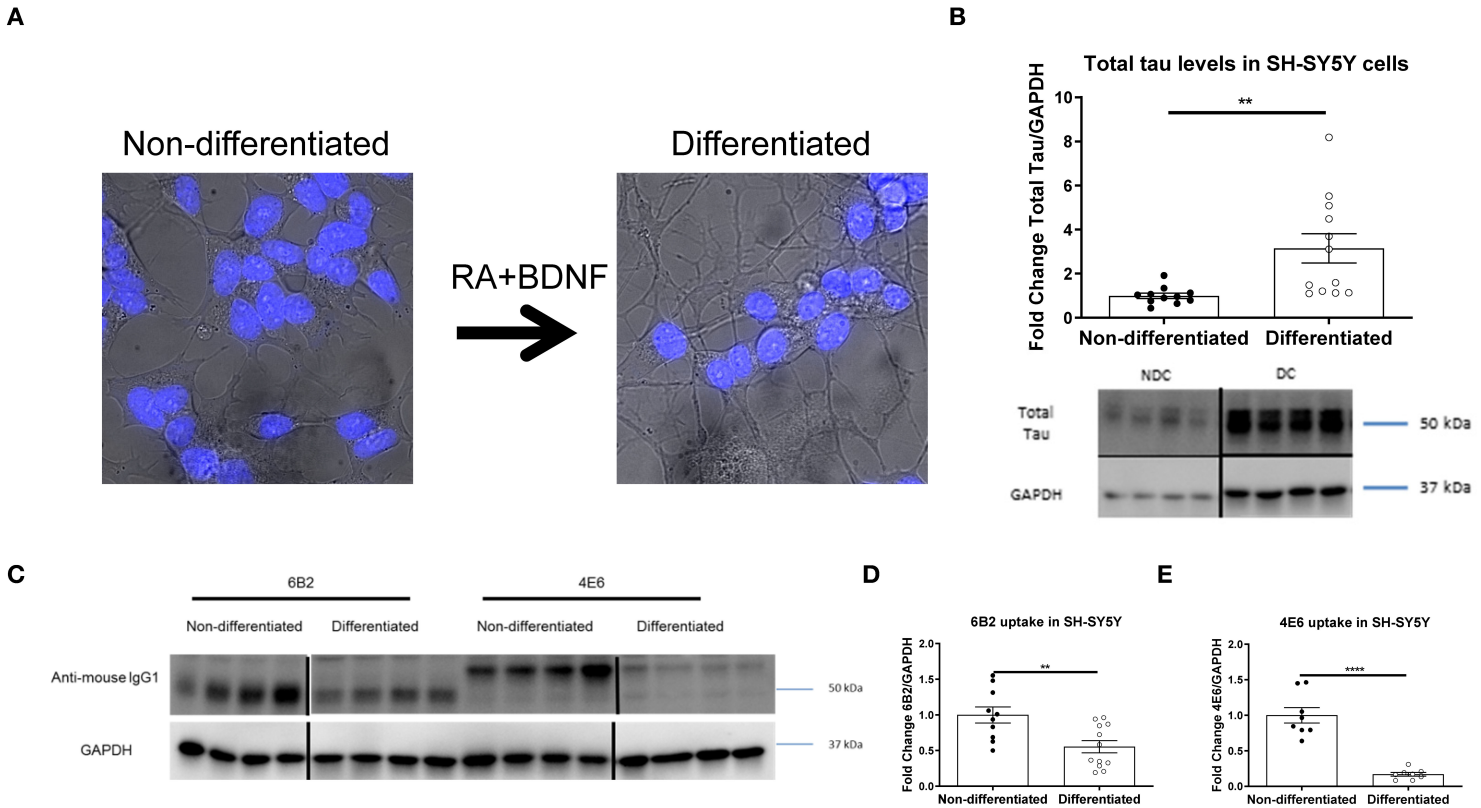
Double differentiation transforms SH-SY5Y cells into a more neuronal-like state, with increase in tau levels and decrease in antibody uptake. Differentiated and non-differentiated SH-SY5Y cells (DC and NDC) were analyzed using live imaging microscopy, or lysed and collected for Western blot analyses. **(A)** Shows live imaging of NDC and DC from the bright-field channel, with nuclei in blue. DC have smaller cell bodies, and longer processes than their NDC counterparts. **(B)** Shows representative total tau and GAPDH Western blots of both cell types with quantification of total tau normalized to GAPDH. DC have significantly more total tau protein than NDC (215% increase, **p = 0.006, *n* = 11–12, *t*-test, two-tailed). DC and NDC were also treated with tau antibodies (6B2 and 4E6) for 24 h. Cells were then lysed and collected for Western blot analyses, and probed with an anti-mouse IgG1-HRP to measure antibody uptake. Anti-mouse IgG1 signal was normalized to GAPDH. **(C)** Shows representative Western blots of NDC and DC samples where the IgG1 antibodies (6B2 and 4E6) appear to have slightly different weights, which may be due to their slightly different isoelectric points (~6.8 and ~6.5, respectively), and may explain why they run on the gel differently. **(D,E)** Shows the quantified results of the Western blots. Uptake of the 6B2 tau antibody (45% reduction, **p = 0.005, *n* = 10–12, *t*-test, two-tailed) and the 4E6 tau antibody (83% reduction, ****p < 0.0001, *n* = 8, *t*-test, two-tailed) were less in DC compared to NDC. Note that the blot lanes in **(B,C)** are from the same blots, respectively. The lines show where excess test lanes where sectioned out. All scatter bar graphs are mean ± SEM.

In prior studies we used several different modeling approaches to examine the efficacy and mechanism of action of tau antibodies. The tau antibodies used herein were generated against Tau386-408[P-Ser396, 404], a prominent pathological epitope in AD and other tauopathies, and have been characterized previously to some extent, showing the efficacy of the 4E6 antibody and relative lack thereof for the 6B2 antibody (7, 12, 14, 16, 19, 22, 23, 27). To better understand the mechanism of action and further clarify the dynamic interaction between tau antibodies and pathological tau, we expanded our research toward alternative culture models, focusing initially on non-differentiated SH-SY5Y cells (NDC), which with the help of flow cytometry provided quantitative insight into uptake and colocalization of the antibodies and their target (19). To improve this model by rendering it more neuron-like, we differentiated these cells with retinoic acid and BDNF as previously described (23, 28–31, 35–37). As expected, differentiated cells (DC) had smaller cell bodies, and elongated processes (Figure 1A) compared to NDC, as well as increased total tau levels on Western blots (215% increase, *p* < 0.01, Figure 1B). To clarify that DC could take up tau antibodies, both types of cells were treated with the 6B2 or 4E6 tau antibody for 24 h and then analyzed on Western blots, which revealed that DC took up less 6B2 or 4E6 than NDC (6B2; 45% reduction, *p* < 0.01, Figures 1C,D; 4E6; 83% reduction, *p* < 0.0001, Figures 1C,E). We then showed the efficacy of the 4E6 antibody in preventing toxicity of human brain-derived paired helical filament (PHF)-enriched tau protein in DC, whereas it was ineffective in NDC, presumably because the latter model does not have a fully developed clearance system (Supplementary Figure 1). The 6B2 tau antibody was ineffective in both models and neither antibody was toxic on their own (Supplementary Figure 1). PHF cytotoxicity is associated with decreases in NeuN levels on Western blots as described previously by us (12, 23) and others (35, 38–42).

### Differentiated SH-SY5Y Cells Express a Different Repertoire of Fc Receptors Compared to Non-differentiated Cells

**Figure 2:**
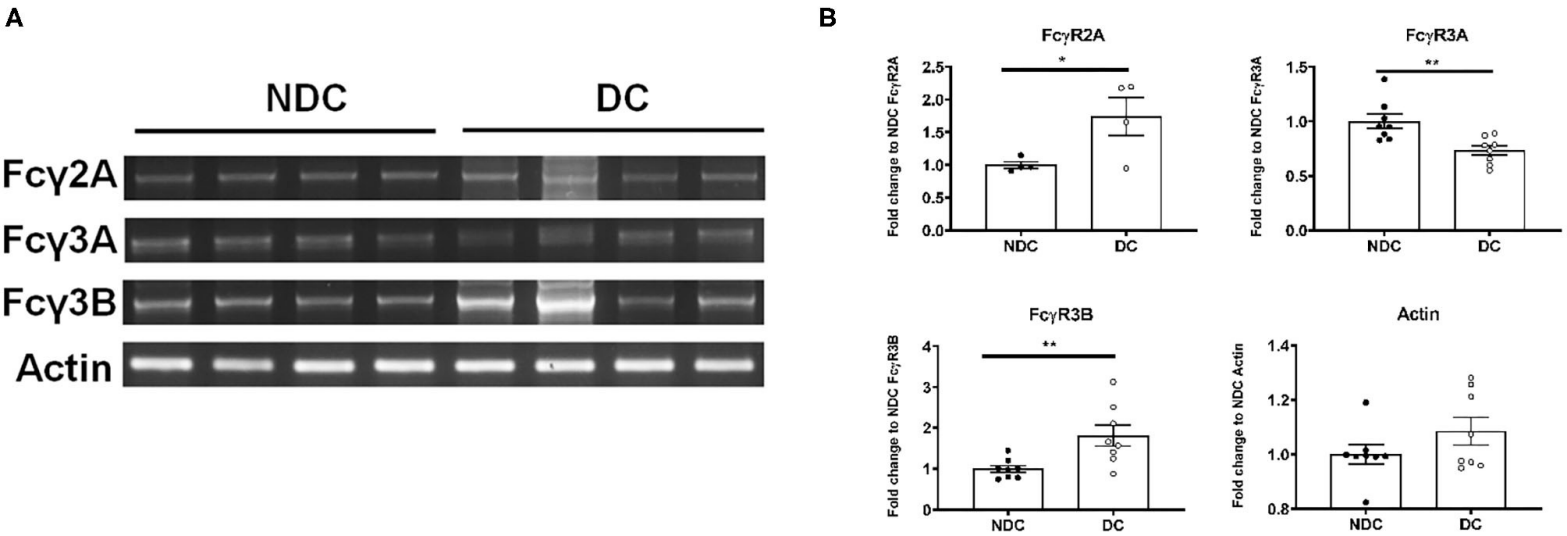
Subtypes of Fcγ receptors are transcribed in NDC and DC. NDC and DC were kept at basal conditions as described in Figure 1. Cells were lysed, and RNA was extracted. Total RNA was then subject to reverse transcriptase to generate cDNA, and it subsequently amplified by PCR with FcγR2A, FcγR3A, FcγR3B, and actin primers. All amplified samples (FcγR2A = 3,894 base pairs (bp), FcγR3A = 81 bp, FcγR3B = 1,631 bp, Actin = 189 bp. were run on a 2% agarose gel and imaged for quantification and analysis. **(A)** Shows representative mRNA expression of indicated genes on an agarose gel. **(B)** Shows quantification of those gels. DC expressed more of FcγR2A (74% increase, *p < 0.05, *n* = 4, Students *t*-test, two-way) and FcγR3B (82% increase, **p < 0.01, *n* = 8, Students *t*-test, two-way) but less of FcγR3A (27% reduction, **p < 0.01, *n* = 8, Students *t*-test, two-way), compared to NDC. Actin was unchanged between the cell models. All scatter plots are mean ± SEM.

**Figure 3:**
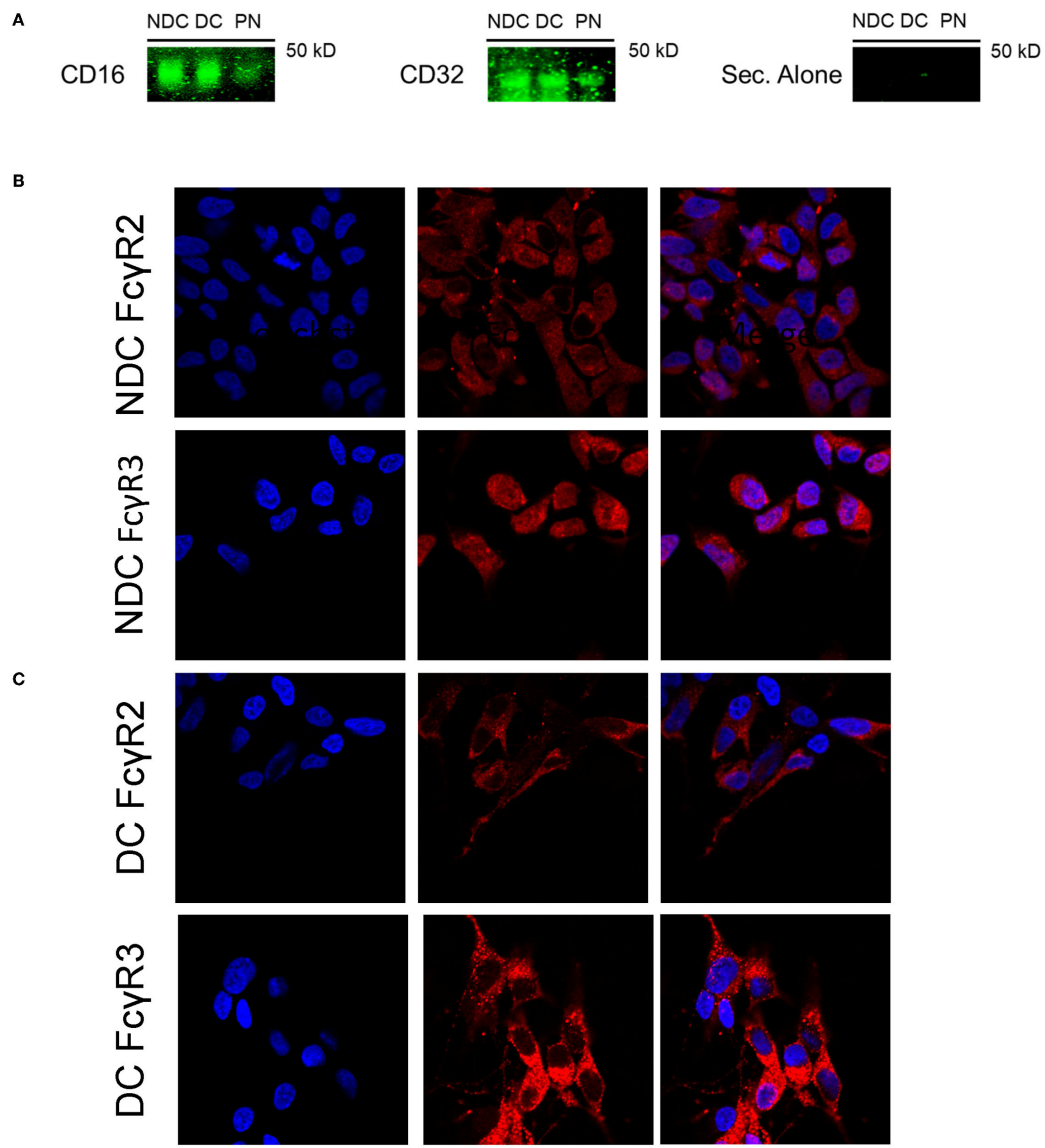
Subtypes of Fcγ receptors are expressed in NDC and DC. Cells were evaluated for presence of FcRs via Western blot and immunohistochemistry. All cells were kept at basal conditions. For Western blot, cells were lysed and equal amounts of protein were added to immunoprecipitation (IP) beads bound to an anti-mouse CD16/32 antibody. Following IP, the samples were subjected to immunoblotting, and the blots probed with antibodies recognizing CD16 and CD32, or fluorescent secondary alone (Sec. Alone). **(A)** Using both antibodies, the signal is similar in the undifferentiated and differentiated neuroblastoma cells. A band is visible in the lane containing primary neuron samples indicating the presence of receptors on these cells. No signal was visible when the blot was probed with only the fluorescent secondary antibody. For immunohistochemistry, cells were fixed, washed, stained for surface bound Fcγ receptors (FcγR); CD16 (FcγR3) and CD32 (FcγR2), and then imaged using confocal microscopy. Cell images were enlarged further 4x from original file size. **(B,C)** Analysis of images revealed diffuse staining of both FcγR in or near the soma for DC and NDC. DC showed staining as well in distal processes and neurites, which are not developed in NDC.

In prior studies in mouse neurons, we concluded that Fcγ receptors (FcγR) were the primary mechanism of uptake of tau antibodies because it could be blocked by Dynasore, an inhibitor of clathrin (receptor)-mediated endocytosis and by an antibody against Fcγ2/3 receptors (mouse BD Fc Block™) (14). We first confirmed that Dynasore prevented antibody uptake in DC, whereas it was ineffective in NDC because its uptake is primarily bulk-mediated as supported by dextran-colocalization studies (Supplementary Figure 2). We did not test the Fcγ2/3 blocking antibody because it is made for mouse receptors and therefore unlikely to work in this human culture model. To clarify if these same receptors were expressed in SH-SY5Y cells, we measured their mRNA expression of Fcγ receptors (FcγR) under basal conditions using PCR and quantitative analysis. DC expressed more of FcγR2A (74% increase, *p* < 0.05), and FcγR3B (82% increase, *p* < 0.01), and less of FcγR3A (27% reduction, *p* < 0.01), compared to NDC, with actin levels being comparable in both models (Figures 2A,B). Expression of FcγR3A and B was detected consistently in different batches of cells, whereas FcγR2A was not detected in all batches. FcγR2 and FcγR3 expression was further confirmed by immunoprecipitation/western blots and confocal microscopy. FcγR2 (CD32) and FcγR3 (CD16) protein was detected in equal amounts in both cells types via Western blot following immunoprecipitation (Figure 3A). All FcγR were localized at the cell body for NDC (Figure 3B), as they have no processes, while they could be visualized in the cell body and distal processes for DC (Figure 3C). These different expression levels, in addition to other mechanistic differences (Supplementary Figure 2), may explain why NDC and DC take up tau antibodies to different degrees.

### Long-Term Tau Antibody Treatment Prevents Neuro- and Synaptotoxicity in Differentiated Cells

**Figure 4:**
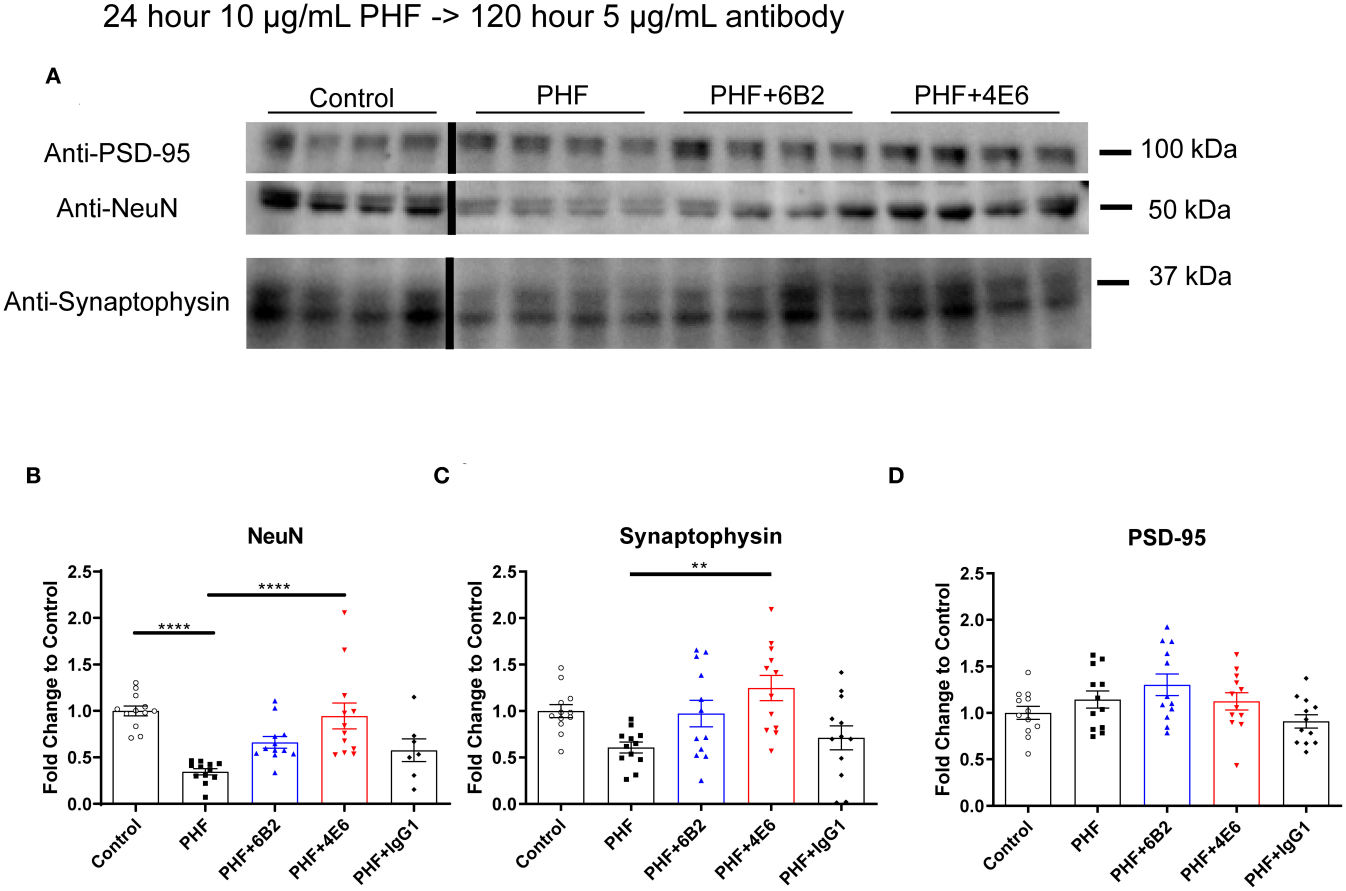
4E6 tau antibody prevents cytotoxicity and synaptotoxicity in PHF pre-treated DC. To further promote a pathological state and clarify antibody efficacy following long-term treatment, DC were pre-treated with higher PHF concentration (10 μg/mL) for 24 h, washed with DMEM, and subsequently treated with 6B2, 4E6, or IgG1 for 120 h (*n* = 7–12 per group). Cells were then lysed and collected for Western blot analyses, and probed for NeuN, synaptophysin, and PSD-95. **(A)** Shows representative Western blots of DC treated with 10 μg/mL PHF and/or 5 μg/mL antibodies, compared to untreated controls. **(B)** PHF (10 μg/ml) was cytotoxic (65% reduction in NeuN levels, ****p < 0.0001, One-way ANOVA, Bonferroni *post hoc* test). Subsequent 4E6 incubation for 120 h significantly increased NeuN levels back to control levels (****p < 0.0001), while 6B2 and IgG1 had no effect. **(C)** Likewise, PHF showed a strong trend for synaptotoxicity (39% decrease in the presynaptic marker synaptophysin). Subsequent 4E6 incubation for 120 h significantly increased synaptophysin levels compared to the PHF group (106% increase, **p < 0.01, One-way ANOVA, Bonferroni *post hoc* test), while 6B2 and IgG1 had no effect. **(D)** The postsynaptic marker PSD-95 was not altered overall or between the groups. Note that the blot lanes in the individual blots are from the same blot. The lines show where excess test lanes where sectioned out. All scatter bar graphs are mean ± SEM.

To further clarify PHF toxicity and antibody efficacy, DC were treated with PHF (10 μg/mL), which creates a more homogenous pathological condition than lower doses (Supplemental Figure 3), for 24 h. Cells were subsequently treated with antibodies (5 μg/mL) for 120 h, followed by Western blot analyses (Figure 4A). The groups differed overall (*p* < 0.0001). PHF was clearly toxic as assessed by the decreased NeuN levels at 144 h compared to untreated control (65% reduction, *p* < 0.0001, Figure 4B). The 4E6 tau antibody prevented PHF toxicity as NeuN levels were similar to untreated control values (*p* < 0.0001), compared to the PHF group. There was a trend for the 6B2 tau antibody to prevent PHF toxicity (*p* = 0.101), whereas IgG1 had no effect on PHF toxicity (*p* = 0.988) (Figure 4B). In addition, synaptophysin and PSD-95, which are pre- and post-synaptic markers, were measured to assess synaptic integrity. Synaptophysin levels differed overall between the groups (*p* < 0.0016). They trended downward due to PHF toxicity (39%, Figure 4C), and 4E6 prevented this as following its incubation this marker went back to untreated control levels (*p* = 0.0018), compared to the PHF group. There was a trend for the 6B2 tau antibody to prevent this type of PHF toxicity (*p* = 0.257), whereas IgG1 had no effect on synaptophysin (*p* > 0.999). Lastly, the post-synaptic marker PSD-95 was not significantly affected by PHF or subsequent antibody treatments (Figure 4D). Briefly, these findings are in line with our prior work showing efficacy of 4E6 and lack thereof for 6B2 in preventing tau toxicity in primary mouse neurons and in a transgenic mouse model (12). Hence, these findings validated the use of this DC model for subsequent live imaging studies.

### 4E6 Tau Antibody Is Taken Up Into the Soma of Differentiated Cells Whereas 6B2 and IgG1 Enter via Neuronal Processes as Assessed by Live Imaging

**Figure 5:**
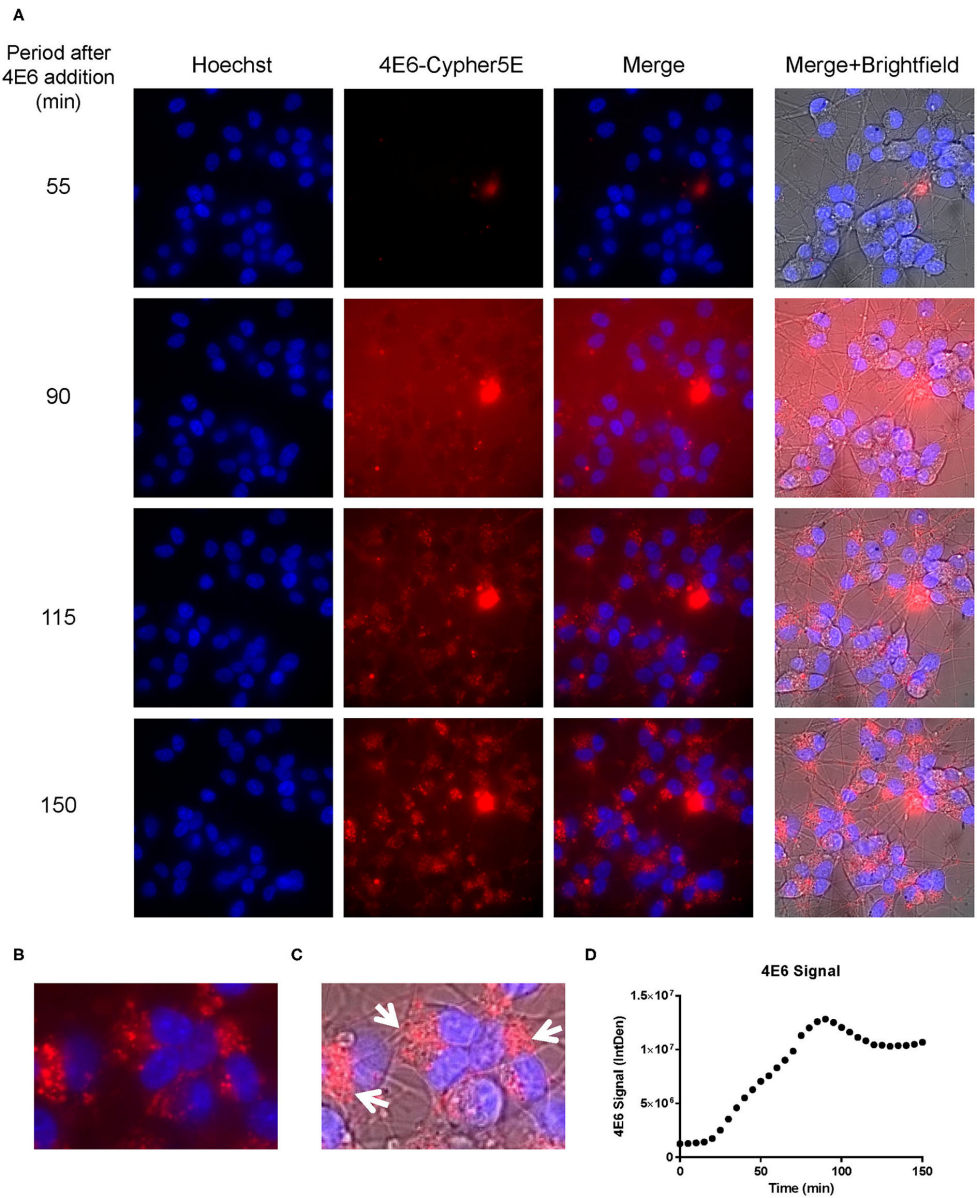
4E6 tau antibody is time-dependently taken up into the soma of DC in a saturable process. Prior to imaging, DC were incubated with a nuclear stain, Hoechst, washed, and then placed in non-phenol red DMEM with HEPES. Subsequently, DC were treated with 20 μg/mL Cypher5E (a pH sensitive dye)-tagged 4E6 tau antibody for up to 140 min. Cells were analyzed using live time-lapse imaging at 5 min intervals. Time points for analyses were chosen from the first time point with positive antibody signal. **(A)** Shows representative still images from the live imaging from the 55 to 150 min time points, with all analyzed channels. The 4E6-Cypher5E signal increased over time as the antibody enters more acidic compartments within the cell (endosomes to lysosomes). At 90 min there was increased background noise, due to the relatively low antibody signal, but it was gone near 100 min. **(B,C)** The 150 min Merge + Brightfield images were magnified to show more detailed morphology and localization of the 4E6 tau antibody in the cells. Most of the antibody signal was localized in the soma of the cells, and was peri-nuclear as indicated by the white arrows. **(D)** Quantification of the 4E6 tau antibody signal from the Cy5 channel from the Internal Density (IntDen) of the entire frame from 0 to 150 min. The 4E6 signal increased over time, and plateaued near 90–100 min.

**Figure 6:**
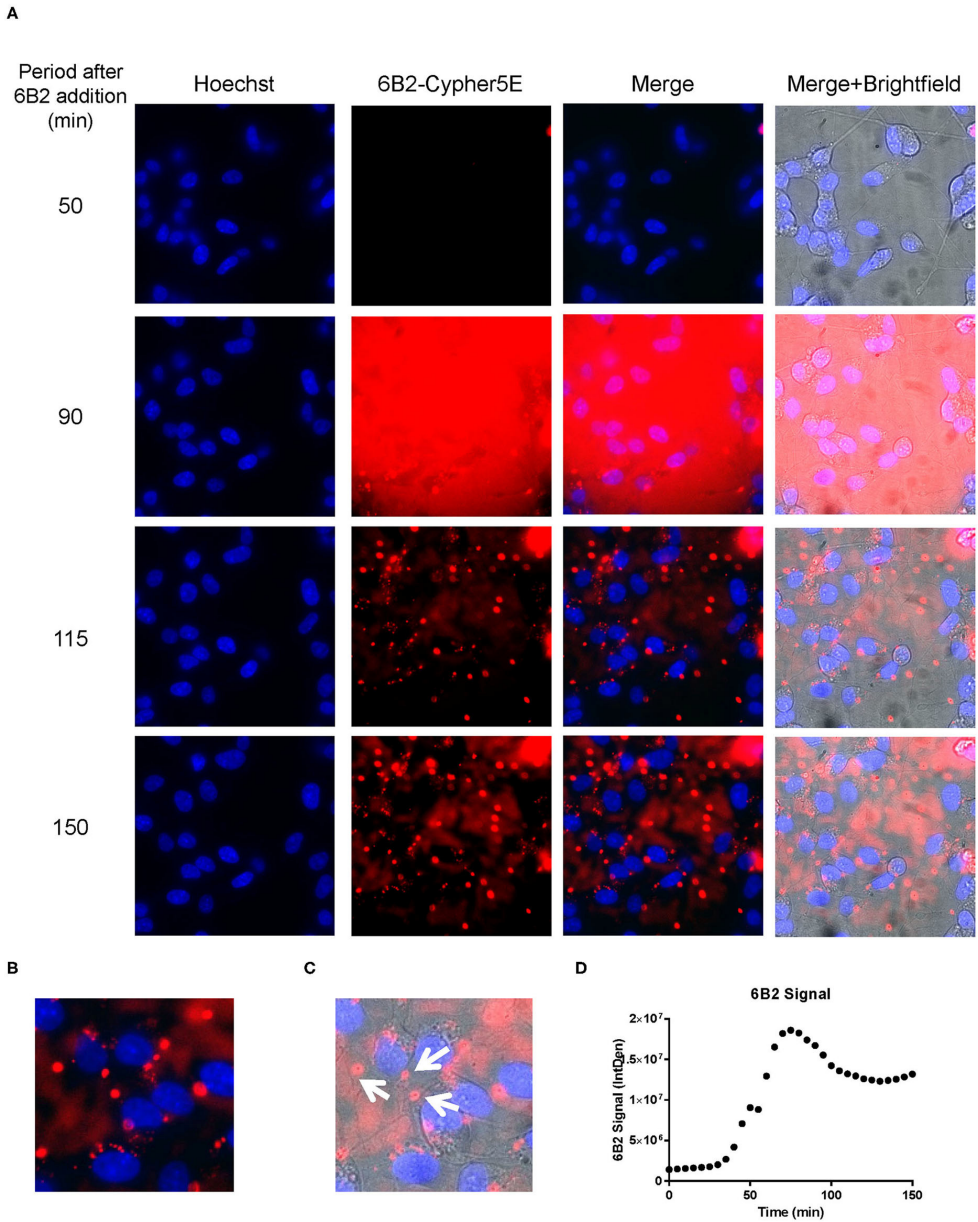
6B2 tau antibody is time-dependently taken up into the neurites of DC. Prior to imaging, DC were incubated with a nuclear stain, Hoechst, washed, and then placed in non-phenol red DMEM with HEPES. Then, DC were treated with 20 μg/mL Cypher5E-tagged 6B2 tau antibody for up to 150 min. Cells were analyzed using live time-lapse imaging at 5 min intervals. Time points for analyses were chosen from the first time point with positive antibody signal. **(A)** Shows representative still images from the live imaging from the 50 to 150 min time points, with all analyzed channels. The 6B2-Cypher5E signal increased over time. At 90 min there was increased background noise, due to the relatively low antibody signal, but it was gone near 100 min. **(B,C)** The 150 min Merge + Brightfield images were magnified to show more detailed morphology and localization of 6B2 tau antibody in the cells. Most of the antibody signal was localized in the neurites of the cells as indicated by the white arrows. **(D)** Quantification of the 6B2 tau antibody signal from the Cy5 channel from the Internal Density (IntDen) of the entire frame from 0 to 150 min. The 6B2 signal increased over time, and plateaued near 105 min.

**Figure 7:**
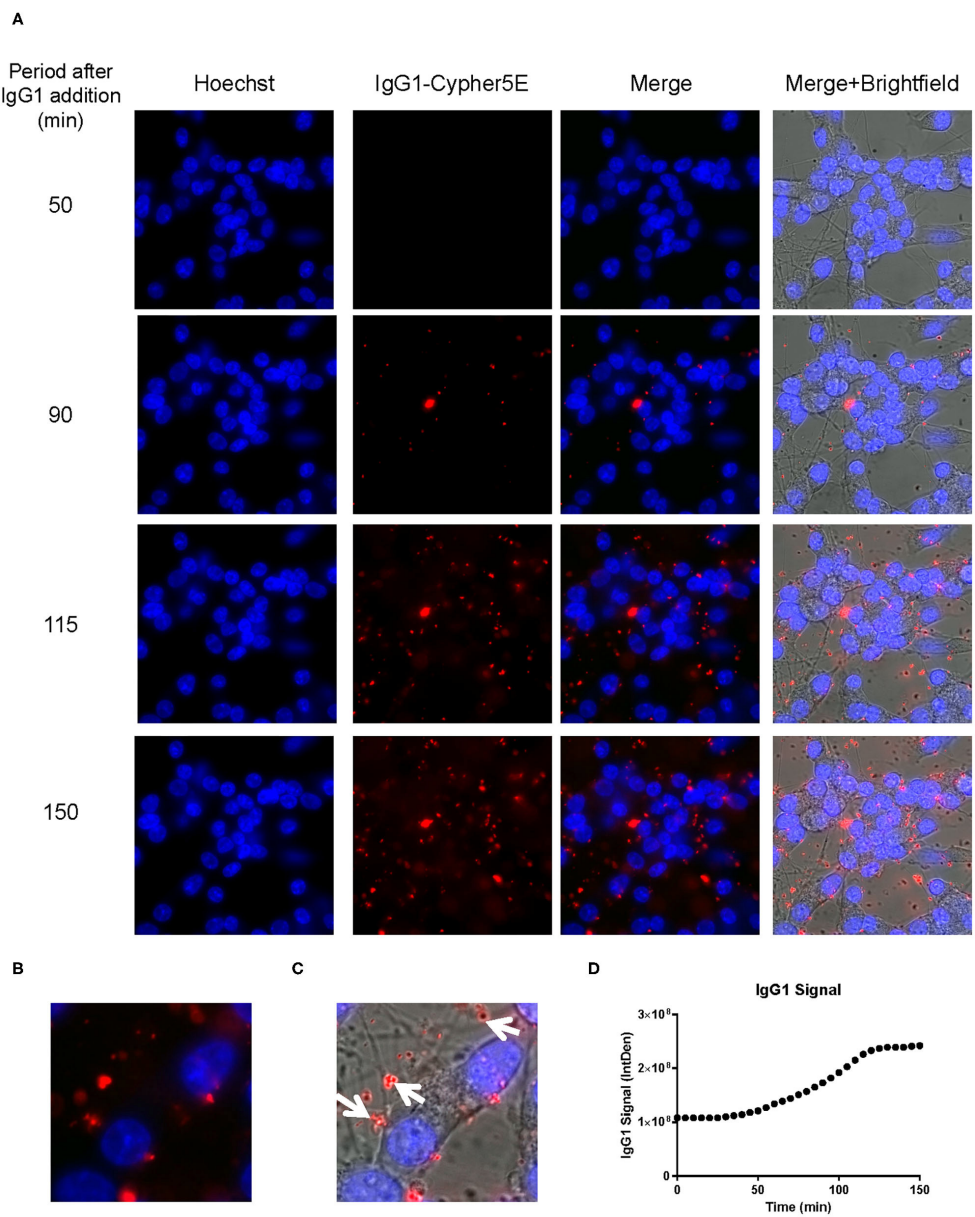
IgG1 antibody is time-dependently taken up into the neurites of DC. Prior to imaging, DC were incubated with a nuclear stain, Hoechst, washed, and then placed in non-phenol red DMEM with HEPES. Subsequently, DC were treated with 20 μg/mL Cypher5E-tagged IgG1 antibody for up to 150 min. Cells were analyzed using live time-lapse imaging at 5 min intervals. Time points for analyses were chosen from the first time point with positive antibody signal. **(A)** Shows representative still images from the live imaging from the 50 to 150 min time points, with all analyzed channels. The IgG1-Cypher5E signal increases over time. **(B,C)** The 150 min Merge + Brightfield images were magnified to show more detailed morphology and localization of 4E6 tau antibody in the cells. The greater majority of the antibody signal was localized in the neurites of the cells as indicated by the white arrows. **(D)** Quantification of the IgG1 antibody signal from the Cy5 channel from the Internal Density (IntDen) of the entire frame from 0 to 150 min. The IgG1 signal increased over time, and plateaued near 120 min.

We had previously shown that both the effective 4E6 tau antibody and the ineffective 6B2 tau antibody could localize with somatic intraneuronal tau in mouse brain slices and in mouse primary neurons following an incubation for 24 h or longer. Those findings do not provide insight into why the former is effective and the latter ineffective. Therefore, we set out to determine with live imaging if earlier cellular events might provide better insight into this important issue. DC are preferred over primary neurons for live imaging as they are less sensitive to the conditions for such analysis. The DC were treated for up to 150 min with 4E6 or 6B2 tau antibodies or control IgG1 (20 μg/mL), which were tagged with a pH sensitive dye (Cypher5E). This is an ideal dye as it is only detectable in acidic compartments within the cell. Its signal therefore confirms intracellular location as opposed to extracellular detection. Larger amounts of antibodies were used than in prior experiments to enhance detection by live imaging under the shorter time period. Antibody internalization was monitored using time-lapse live imaging, which revealed that antibody uptake was time-dependent (Figure 5A). 4E6 tau antibody signal was primarily detected in the soma, and was peri-nuclear (Figures 5B,C). In addition, the signal began to plateau near 90 min after incubation began (Figure 5D), suggesting a saturable process. Both 6B2 tau antibody and IgG1 were also taken up in a time-dependent manner (Figures 6A, 7A), and distinctly localized in the neurites and more distal processes of the DC, with limited visibility in the soma (Figures 6B,C, 7B,C). 6B2 signal increased over time and plateaued near 100 min, while IgG1 did not have as a distinct plateau over the same period (Figures 6D, 7D), suggesting that its uptake may not have reached capacity. These finding indicate that the dynamics of the uptake of the two tau antibodies and its control antibody are different and needed to be examined further for their interaction with pathological tau protein for a possible insight into their efficacy or lack thereof.

### Live Imaging Reveals Dynamics of 4E6 Co-localization With PHF in Differentiated Cells

**Figure 8:**
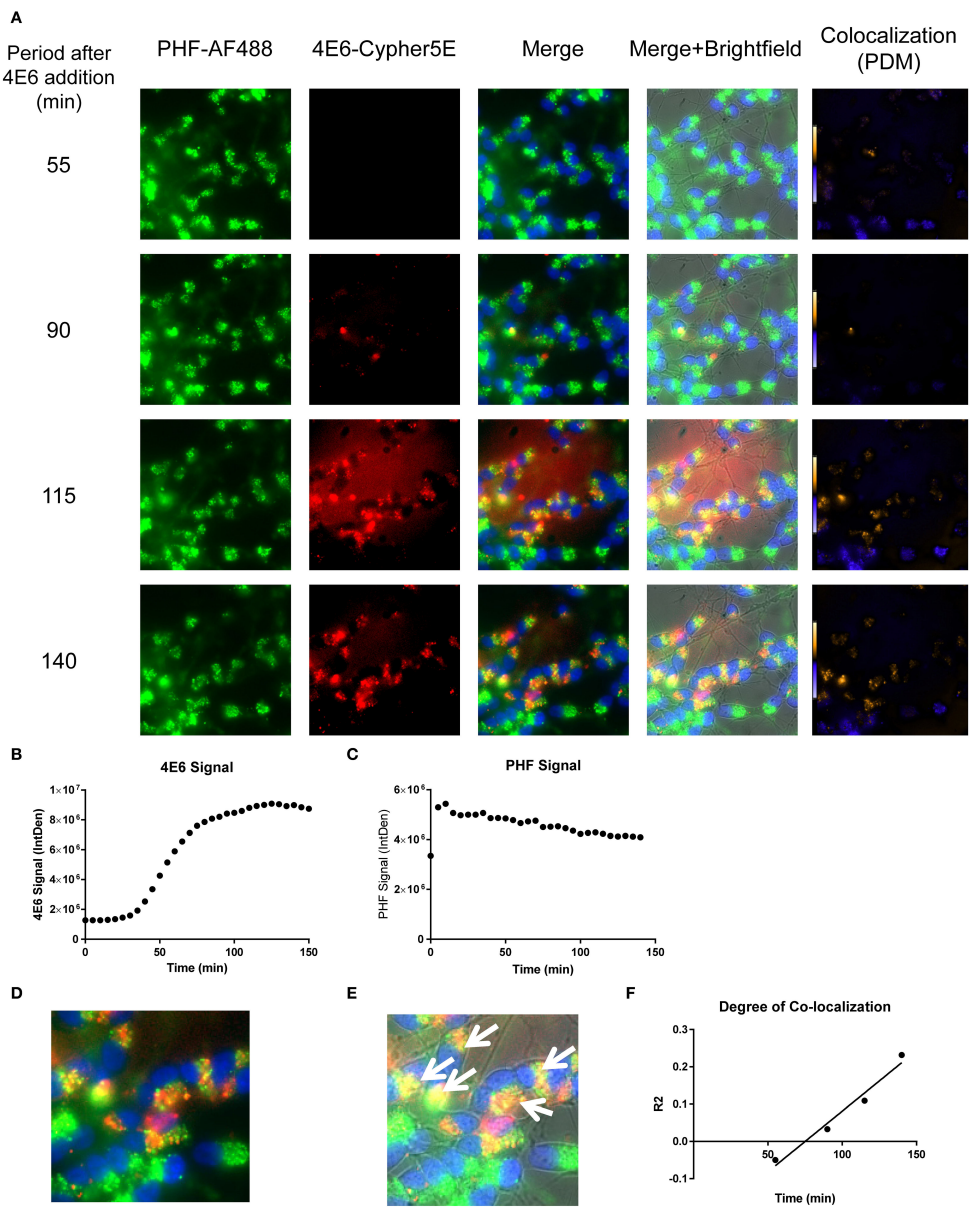
Time-lapse live imaging shows a strong correlation between PHF and 4E6 in pre-treated DC. DC were pre-incubated with 50 μg/mL tagged PHF-AF488 for 16 h, washed, incubated with Hoechst stain, washed again, and then incubated with Cypher5E-tagged 4E6 tau antibody for up to 140 min. Cells were analyzed using live time-lapse live imaging at 5 min intervals. **(A)** Shows still frames from the live imaging of the 55–140 min time points, with all analyzed channels. The 4E6-Cypher5E signal increased over time, while the PHF signal did not change. In addition, an intensity co-localization analyses was performed between the 4E6 and PHF signals, which generates a co-localization heat map and intensity correlation coefficient (*R*^2^). The colocalization heat map (PDM) showed increasing intensity over time, as indicated by the yellow color, which depicts greater colocalization. Internalization of 4E6 occurred primarily in the soma, and was peri-nuclear, while the PHF resided in the soma at the beginning of the experiment. **(B)** Shows quantification of the 4E6-Cypher5E signal, where the signal increased over time, and began to plateau near 75 min. **(C)** Shows quantification of the PHF-AF488 signal, which showed no change within the 140 min. **(D,E)** The 140 min Merge and Merge + Brightfield images were magnified to show more detailed morphology and localization of 4E6 tau antibody and PHF co-localization in the cells. Most of the antibody signal was localized in the soma of the cells, and was peri-nuclear as indicated by the white arrows. **(F)** Shows the intensity correlation coefficients of PHF and 4E6 (*R*^2^ = −0.05 to 0.232), which increased linearly, and correlated well over time (*r*^2^ = 0.969, *p* = 0.0157, Pearson, two-tailed).

**Figure 9:**
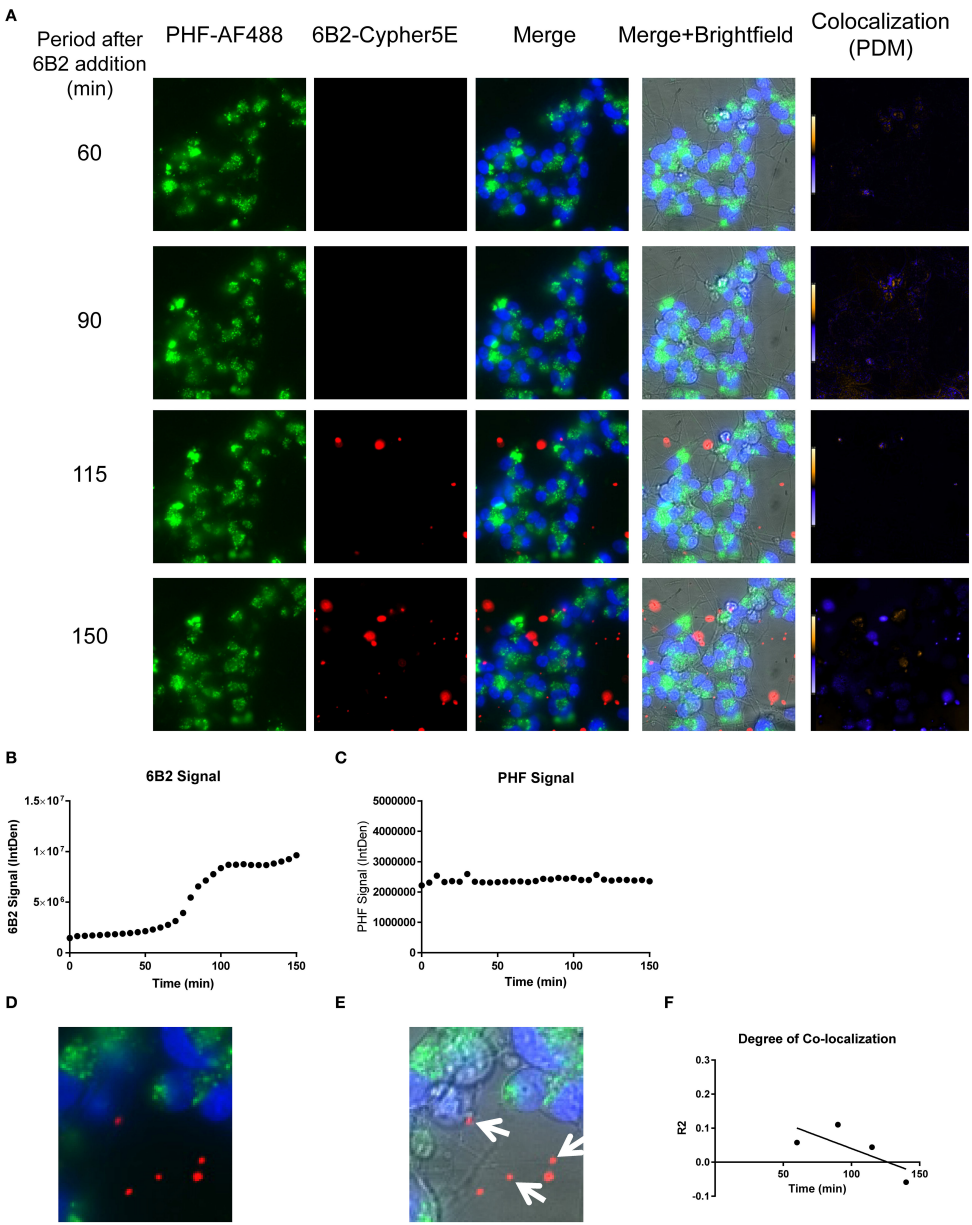
Time-lapse live imaging shows PHF and 6B2 in pre-treated DC, but in separate compartments. DC were pre-incubated with 50 μg/mL tagged PHF-AF488 for 16 h, washed, incubated with Hoechst stain, washed again, and then incubated with Cypher5E-tagged 6B2 tau antibody for up to 150 min. Cells were analyzed using live time-lapse imaging at 5 min intervals. **(A)** Shows still frames from the live imaging of the 55–150 min time points, with all analyzed channels. The 6B2-Cypher5E signal increased over time, while the PHF signal did not change. In addition, an intensity co-localization analyses was performed between the 6B2 and PHF signals, which generates a co-localization heat map and intensity correlation coefficient (*R*^2^). The colocalization heat map (PDM) showed no change in intensity over time. Internalization of 6B2 occurred primarily in the neurites, while the PHF resided in the soma. **(B)** Shows quantification of the 6B2-Cypher5E signal, where the signal increased over time, and began to plateau near 100 min. **(C)** Shows quantification of the PHF-AF488 signal, which showed no change within the 150 min period. **(D,E)** The 150 min Merge and Merge + Brightfield images were magnified to show more detailed morphology and localization of 6B2 tau antibody and PHF in the cells. Most of the antibody signal was localized in the neurites of the cells, and did not colocalize with PHF, as indicated by the white arrows. **(F)** Shows the intensity correlation coefficients of PHF and 6B2 (*R*^2^ = 0.058 to −0.059), which did not correlate over time (*r*^2^ = 0.5334).

**Figure 10:**
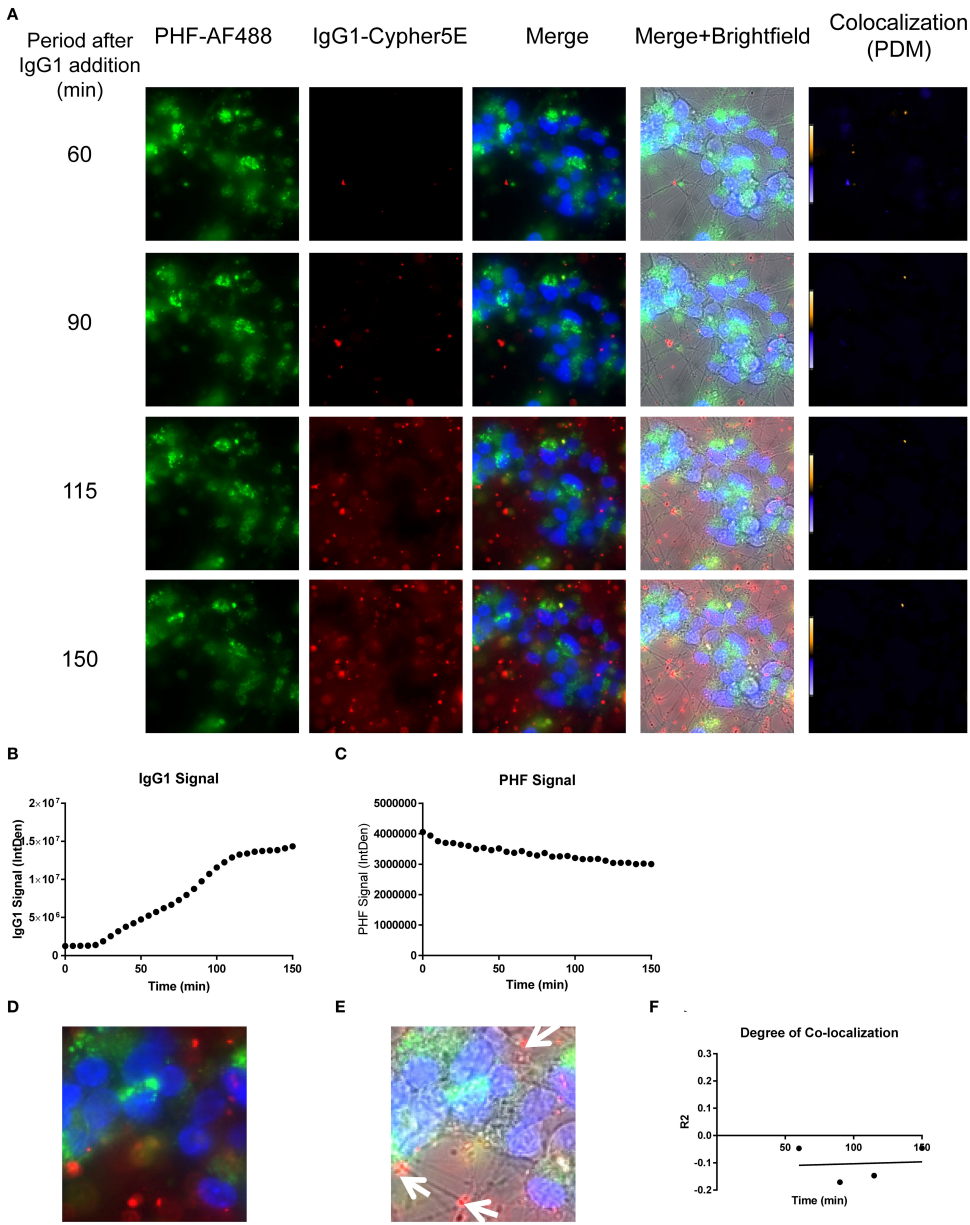
Time-lapse live imaging shows PHF and IgG1 in pre-treated DC, but in separate compartments. DC were pre-incubated with 50 μg/mL tagged PHF-AF488 for 16 h, washed, incubated with Hoechst stain, washed again, and then incubated with Cypher5E-tagged IgG1 isotype control antibody for up to 150 min. Cells were analyzed using live time-lapse imaging at 5 min intervals. **(A)** Shows still frames from the live imaging of the 55–150 min time points, with all analyzed channels. The IgG1-Cypher5E signal increased over time, while the PHF signal did not change. In addition, an intensity co-localization analyses was performed between the IgG1 and PHF signals, which generates a co-localization heat map (PDM) and intensity correlation coefficient (*R*^2^). The colocalization heat map showed no change in intensity over time. Internalization of IgG1 occurred primarily in the neurites, while the PHF resided in the soma. **(B)** Shows quantification of the IgG1-Cypher5E signal, where the signal increased over time, and began to plateau near 110 min. **(C)** Shows quantification of the PHF-AF488 signal, which showed no change within the 150 min period. **(D,E)** The 150 min Merge and Merge + Brightfield images were magnified to show more detailed morphology and localization of IgG1 and PHF in the cells. Most of the antibody signal was localized in the neurites of the cells, and did not colocalize with PHF, as indicated by the white arrows. **(F)** Shows the intensity correlation coefficients of PHF and IgG1 (*R*^2^ = −0.047 to −0.046), which did not correlate over time (*r*^2^ = 0.0069).

We have previously shown that added PHF-tau is readily detected intracellularly in NDC and in primary mouse neurons after 24 h incubation, and that it takes at least 48 h for it to result in measurable toxicity (12, 19). Likewise, in these models, treatment with tau antibodies for 24 h results in robust intraneuronal colocalization with the PHF. To clarify the earliest interaction between PHF and tau antibodies under the time-lapse live imaging conditions described above, DC were pre-incubated with 50 μg/mL Alexa Fluor 488 tagged-PHF for 16 h, and were subsequently treated with 20 μg/mL Cypher5E tagged-4E6, 6B2, or IgG1 isotype control antibodies for up to 150 min (Figures 8–10, See Supplementary Videos 1–3 for detailed view). Larger amounts of PHF and antibodies were used than in prior experiments to enhance detection by live imaging under the shorter time conditions. Antibody and PHF internalization was closely monitored using time-lapse live imaging. These images showed an increase over time of all antibodies' signal (Figures 8A, 9A, 10A), which began to plateau at 75, 100, and 110 min for 4E6, 6B2, and IgG1, respectively (Figures 8B, 9B, 10B). Note that the cells had been pretreated with PHF-tau, and the PHF signal stayed relatively constant for up to 150 min for all antibodies (Figures 8C, 9C, 10C).

Importantly, co-localization with PHF was primarily found within the soma for 4E6, similar to the live imaging results with 4E6 alone (Figure 5), and increased over time (Figures 8A,D,E). 6B2 and IgG1 did not colocalize with PHF, as the antibody signal was primarily within the neurites, similar to the live imaging with those antibodies alone (Figures 6, 7), whereas the PHF was mainly in the soma (Figures 9A,D,E, 10A,D,E). This was further confirmed by colocalization analysis (*R*^2^), which increased rapidly for 4E6 and showed a strong positive correlation over time (*r*^2^ = 0.969, *p* = 0.0157, Figure 8F), but poor or non-existent correlation for 6B2 and IgG1 over time (Figures 9F, 10F). These pronounced internalization and localization differences fit nicely with the efficacy divergence between the two tau antibodies, and indicate that this type of live imaging is helpful to identify promising therapeutic tau antibodies.

## Discussion

Tau immunotherapies have been shown to be effective in various models (1–4). Despite these advances, mechanistic and dynamic insight into the uptake and interaction of pathological tau protein and tau antibodies has been relatively limited. Here, we show that differentiated human SH-SY5Y neuron-like cells (DC) treated with human brain-derived pathological tau, is a suitable and expeditious alternative model to assess and clarify tau antibody efficacy, compared to the more time consuming and more expensive to maintain *in-vivo* and induced pluripotent stem cell models. It is well documented that differentiated SH-SY5Y cells (DC) have improved expression of neuronal markers and morphology compared to their non-differentiated (NDC) counterparts (28–30). This DC model is more scalable than both primary mouse neurons and stem cell models, In addition, live time-lapse imaging of this model is more feasible than in those other culture models, and it provides a valuable temporal and spatial insight into the interaction of pathological tau and potential antibody therapies. Specifically, live imaging can be used as a rapid means of analysis of an antibody's: (a) effectiveness to enter neurons; (b) colocalization with its target; (c) subcellular location, and; (d) its association with its target over time.

To create a more physiological cell culture system, a double-differentiated protocol was employed, as described previously by us (23, 31, 36, 37), and other groups (28–30), to differentiate SH-SY5Y neuroblastoma cells with increased neuronal expression and morphology These cells are characteristically different from their NDC counterparts with: (1) improved neuronal morphology; (2) decreased antibody uptake; and (3) increased tau levels (Figure 1). In addition, we showed that the neuronal marker NeuN is a sensitive way to measure PHF toxicity in these models, as we had previously shown in primary mouse neurons (12, 23), and others have used as well to assess neurotoxicity (35, 38–42). We have previously discussed that NeuN is expressed throughout the neuron, and that the PHF toxicity likely reflects more retraction of neuronal processes than overt cell death within the analyzed time period (12). PHF (1 μg/mL) was cytotoxic to both human cell lines as measured after 48 h incubation as NeuN levels decreased by 53 and 37%, in NDC and DC, respectively. Importantly, 4E6 tau antibody treatment in PHF pre-incubated DC prevented PHF toxicity, but it was ineffective in NDC (Supplementary Figure 1). These DC results are in agreement with findings in primary mouse neuronal cultures under similar conditions (14).

Several human and mouse subtypes of FcγRs exist and are found on various cell types (43). Likewise, several human and mouse IgG isotypes exist with different functions (43). The nomenclature of these receptors and isotypes is not the same for these species, which complicates their comparison. Briefly, regarding tau antibody therapy studies, it is important to keep in mind that most anti-tau mouse monoclonals are of the IgG1 isotype, like the antibodies in this particular study. However, effector function for microglial and macrophage phagocytosis is mainly confined within mouse IgG2a binding to mouse FcγR1 but is limited for mouse IgG1. In contrast, human IgG1 has a strong phagocytic effector function linked to human FcγR1. These differences are though not relevant in the current neuron-like DC model or in mouse primary neuronal cultures because they do not have phagocytic activity. We have previously demonstrated that blocking mouse FcγR1 or phagocytosis has no effect on IgG1 antibody uptake in mouse brain slices or in mouse primary neuronal culture (14). We have also repeatedly shown that the 4E6 IgG1 mouse monoclonal antibody used herein is readily taken up both in mouse neurons (7, 12, 14, 22, 23) and in human NDC (19) and DC in the present article. In contrast, its human IgG1 Fc chimera (c4E6) is poorly taken up in both mouse primary neurons and in human DC because its charge is very different from the mouse IgG1 isotype (23).

Considering that we have previously linked neuronal uptake of antibodies to FcγR2/3 (7), examining their expression in this model was warranted. The mRNA and protein expression levels of FcγR is generally greater in DC than in NDC (Figures 2, 3), which aligns nicely with the main antibody uptake pathway in DC being receptor-mediated, whereas in NDC it is bulk-mediated (Supplementary Figure 2). Expression of the FcγR3 subtypes was seen consistently in different cell batches but not always for FcγR2. This phenomenon needs to be further examined, and it may depend on cell passage. Lastly, the proposed mechanism of action in DC is in agreement with our prior work, revealing that antibody uptake in primary mouse neuronal cultures is mainly receptor-mediated – presumably FcγR2/3-mediated based on blocking studies - while bulk-mediated endocytosis was involved to a lesser extent (14). For all these reasons, and their more neuronal properties, DC are a better model than NDC for future studies in this field, and compares favorably to other models used/developed by our laboratory. Other groups have reported involvement of FcγR in uptake of antibodies targeting tau and α-synuclein in different models (17, 44).

Furthermore, to enhance tau pathology in DC, we increased the PHF dosage to 10 μg/mL, which also generates a more homogeneous cellular pathology (Supplementary Figure 3). We subsequently treated the cells with antibodies for a longer time period (120 h) to better reflect their efficacy in preventing PHF cytotoxicity. As expected, these experiments showed a more pronounced PHF cytotoxicity as measured by NeuN levels (65%), which was prevented by the 4E6 tau antibody, while 6B2 trended this way, and IgG1 had no effect (Figure 4). This study confirmed the efficacy differences between 4E6, 6B2, and IgG1 control as we have seen *in vivo* and in primary mouse neuronal cells (12, 22), further supporting the predictive validity of this human cellular model. PHF-induced synaptotoxicity as revealed by a decrease in synaptophysin, a presynaptic marker, mirrored PHF-induced reduction in NeuN. Importantly, as for NeuN, synaptotoxicity was prevented with 4E6 tau antibody treatment, but not with 6B2 or IgG1. Interestingly, PHF was not toxic to the post-synapse as reflected by the lack of change in PSD-95 levels following PHF incubation. This is not particularly surprising because postsynaptic degeneration (loss of PSD-95) is a later phenomenon than presynaptic degeneration (loss of synaptophysin) in AD and related models (45–47). In addition, it has been shown that TrkB, a receptor of BDNF, and PSD-95 complex together within the post-synapse (48–51), and when bound to BDNF a positive feedback loop leads to an increase in PSD-95 (52, 53). The SH-SY5Y neuroblastoma cells are differentiated with an artificially high level of BDNF (50 ng/mL) (28–30), which is much lower in the interstitial space of the brain under physiological or pathological states (54). As a result, the BDNF concentration in our DC model may saturate the PSD-95 levels.

The establishment of DC as a suitable model for PHF toxicity and its prevention by tau antibodies paved the way for the live time-lapse imaging studies. Specifically, to explore internalization and interaction of tau antibodies and their pathological targets in the immediate aftermath of adding these reagents to the cultures. Our findings indicate that their internalization and subsequent interaction occurs in different sub-cellular locations, depending on the antibody used. In cells treated only with the 4E6 tau antibody, the antibody was preferentially taken up directly into the soma. Its signal increased over time and plateaued at about 90 min, suggesting a receptor-mediated process (Figure 5). As shown in Figures 3B,C, the soma has high density of Fc receptors. For the 6B2 tau antibody and IgG1, uptake into the cells was less prominent and internalization was more extensive in the processes compared to 4E6 (Figures 6, 7). All three antibodies are likely primarily taken up via receptor-mediated process because it is a saturable process, and considering our prior work on elucidating their uptake mechanisms (7, 12, 14, 16, 19, 22, 23, 27). This careful frame-by-frame examination of the video files indicated that the uptake of the 4E6 antibody was predominantly directly into the soma, whereas in contrast 6B2 and IgG1 showed prominent uptake via the neuronal processes. The different primary path of uptake for 6B2 and control IgG1 under these live imaging conditions provides insight into why they are therapeutically ineffective, as further confirmed under the PHF-pre-treated conditions discussed below.

In the PHF pre-treated cells, 4E6 antibody uptake was fast, while the PHF signal stayed constant. The degree of colocalization increased rapidly over time in a highly correlated fashion (*r*^2^ = 0.969). This indicates a dynamic antibody being directed to a static target (Figure 8, Supplementary Video 1). However, the scenario was different for 6B2 or IgG1, as those antibodies showed minimal if any colocalization with PHF during the incubation period (Figures 9, 10, Supplementary Videos 2, 3). Furthermore, the uptake of these antibodies began to plateau later (100–110 min), compared to 4E6 (75 min). In addition, most of the 6B2 and IgG1 antibodies seemed to reside within the neurites, similar to antibody alone controls, and were not detected immediately in the soma, unlike 4E6. A possible explanation for these differences is that 4E6 taken up via somal Fc receptors binds to PHF there in the endosomal/lysosomal system, and is therefore quickly visualized. On the other hand, 6B2 or IgG1 are either not taken up via that somal pathway or do not interact with PHF there and are therefore recycled out of the cell and not seen until they come in through the processes. Transport via that pathway takes longer leading to delayed uptake signal. As we have shown in previous studies (14), 4E6 is taken up into primary mouse neurons mainly through receptor-mediated endocytosis (~80%), while the remainder is bulk-mediated (~20%). Together, these findings indicate that 6B2 and IgG1 are internalized through the neurites, while 4E6 is primarily internalized via the soma.

In previous studies, we have shown that tau antibodies can enter tauopathy neurons in a mouse brain within 1 h after intracarotid injection (5). In addition, tau antibodies colocalize strongly with neuronal pathological tau when analyzed at 2 h in *ex vivo* mouse slice culture models (7, 13), and at 24 h in primary mouse neurons (12). Our live time-lapse imaging technique is in agreement with these previous studies, as tau antibodies were internalized into neurons within 50 min after treatment. In addition, we demonstrated the convergence of tau antibody and its pathological PHF target within 90 to 115 min. Interestingly though, under the current live imaging conditions, 4E6 but not 6B2 colocalizes with endosomal/lysosomal tau. This clarifies 4E6's efficacy in clearing tau, preventing its toxicity and lack thereof for 6B2 as shown here and previously *in vivo* in a tauopathy mouse model and in primary mouse neurons (12).

Until now, the dynamics behind the efficacy differences between the two antibodies had been unexplained. It cannot relate to isotype because both antibodies are IgG1. Furthermore, both 4E6 and 6B2 have similar overall charge (~6.5 and ~6.8, respectively), reflected in their prominent neuronal uptake as reported previously (7, 12). However, their affinities for various forms of tau differ substantially with 6B2 having much higher affinity for the tau peptide epitope and aggregated tau whereas 4E6 binds primarily to soluble pathological tau (7, 12). Hence, a possible explanation may be that in the PHF pre-treated DC, 6B2 may be binding to extracellular PHF that is present in such small quantities that it does not emit a detectable fluorescent signal. The source of the PHF may be trace amounts that remain after extensive washes following PHF pre-incubation, and/or PHF secreted from the neurons following uptake. This may in effect neutralize the 6B2 and it cannot then interact with more toxic soluble forms of tau within the cell. This 6B2-PHF complex may be preferentially taken up via the neurites, possibly by bulk-endocytosis, as shown previously for recombinant or mouse derived purified tau alone (55–57). As IgG1 does not have an affinity for tau, it is not retained in the soma if it is taken up there, and therefore rapidly recycled out of the cells and not detected. The IgG1 signal within the neurites is likely the result of non-specific uptake as highlighted by the lack of colocalization with PHF throughout the experiment. The possible interaction of 6B2 with trace amounts of extracellular PHF can be seen as an artifact of this model but it may also indicate that extracellular interaction of an antibody with pathological tau neutralizes the antibody so that it cannot prevent intracellular tau toxicity and promote tau clearance.

A more straightforward explanation for the contrasting efficacies of 4E6 and 6B2 relates to the differences in their initial location and uptake within the cell, which is comparable without or with PHF in the system (Figures 5, 6 and 8, 9, respectively). We previously showed that both antibodies are taken up into tauopathy brain slices, with the key difference being that 4E6 primarily colocalized with tau in the endosomal-lysosomal system, whereas 6B2 showed more diffuse cytoplasmic staining (7). Here, only antibodies in the endosomes/lysosomes are visualized and because 6B2 shows minimal colocalization with tau within this degradation system, it likely explains its lack of efficacy in the different culture models, and *in vivo* in promoting tau clearance and preventing its toxicity and related cognitive impairments (12, 22). The endosomal/lysosomal compartment is acidic, the pH of the cytosol is neutral and thus any 6B2 bound to cytosolic tau would not be seen here using the CypHer5 dye, and further investigation into this compartment may be warranted. It should be noted though that we previously reported colocalization of 6B2 and tau within endosome/lysosomes in NDC after 24 h antibody incubation (18). However, as shown here that primitive model system cannot be used to assess efficacies of tau antibodies, presumably because it does not have efficient lysosomal degradation system and likely relies more on exocytosis for clearance. In addition, the high affinity interaction of 6B2 with tau may make it more difficult to degrade the 6B2-tau complex, whereas the lower affinity interaction of 4E6 with tau may promote tau disassembly and facilitate access of lysosomal enzymes to degrade tau. Overall, live imaging of these early events within a few hours allows for rapid prediction of antibody efficacy prior to lengthier studies in other models.

In summary, we have demonstrated that tau antibody 4E6 prevents PHF toxicity in human neuroblastoma DC, while tau antibody 6B2 or control IgG1 control do not, which mirrors our prior findings in other *ex vivo* and *in vivo* mouse models (12, 22). Furthermore, the live imaging herein provides an important new insight into the mechanisms behind this efficacy difference. Time-lapse live imaging in DC up to 150 min showed that the interaction of PHF and 4E6 tau antibody is dynamic and robust, with rapid colocalization within the soma of the cell, whereas this was not seen for 6B2 or IgG1 control. These qualitative results were supported by our quantitative evaluation of the PHF and antibody signals using colocalization analyses. These findings, and the distinct differences between 4E6 vs. 6B2, or IgG1, support the predictive validity of this assay and method of analysis. In particular, this entirely human model is ideal to examine efficacy of humanized antibodies prior to lengthy clinical trials, for which alternative mouse models are likely not appropriate. In conclusion, this overall approach is very useful to clarify the mechanisms of tau immunotherapies, and to evaluate clinical candidate tau antibodies for their effectiveness at entering cells and finding their pathological target.

## Data Availability Statement

The raw data supporting the conclusions of this article will be made available by the authors, without undue reservation.

## Ethics Statement

The use of human tissue in these studies is exempt from Institutional Review Board (IRB) approval as acknowledged by the IRB Committee of the university. Written informed consent for participation was not required for this study in accordance with the national legislation and the institutional requirements. Primary mouse culture generation was approved by the Institutional Animal Care and Use Committee (IACUC) of the university and is in accordance with NIH Guidelines, which meet or exceed the ARRIVE guidelines.

## Author Contributions

DS and ES conceived and designed the experiments, analyzed the data, and wrote the manuscript. DS prepared and performed most of the experiments. YD helped with setup and analysis of data, and wrote relevant method section for live imaging experiments. QW, SM, and EC contributed to the FcR data, determination of the lack of antibody toxicity and relevant text. All authors contributed to the article and approved the submitted version.

## Conflict of Interest

ES is an inventor on patents on tau immunotherapy and related diagnostics that are assigned to New York University. Some of this technology is licensed to and is being co-developed with H. Lundbeck A/S. The remaining authors declare that the research was conducted in the absence of any commercial or financial relationships that could be construed as a potential conflict of interest.

## Supplementary Material

Supplementary Figure 1 4E6 tau antibody prevents PHF toxicity in DC, but not in NDC. NDC or DC, maintained as described above, were pre-treated with PHF (1 μg/mL) for 24 h, washed with DMEM, and subsequently treated with tau antibodies (6B2 or 4E6) for 24 h. Cells were then lysed, collected for Western blot analyses, and probed for NeuN. **(A,B)** Shows representative Western blots of NDC and DC treated with PHF and antibodies, compared to untreated controls. **(A)** Shows the quantified NeuN data from the Western blots for NDC samples, where PHF toxicity decreased NeuN levels (53% decrease, ^**^*p* < 0.001, *n* = 7–8, One-way ANOVA, Bonferroni *post hoc* test). Tau antibodies had no effect in NDC. **(B)** Shows quantified NeuN data for DC samples, where PHF toxicity decreased NeuN levels (37% decrease, ^**^*p* < 0.01, *n* = 12, One-way ANOVA, Bonferroni *post hoc* test), which was prevented by 4E6 resulting in NeuN levels comparable to controls. ^****^*p* < 0.0001 compared to PHF alone. Note that the blot lanes in **(A,B)** are from the same blots, respectively. The lines show where excess test lanes where sectioned out. **(C)** Shows representative Western blots from DC treated with 4E6 and 6B2. Quantitation shows no significant difference observed in the NeuN levels for cells treated with 4E6 and 6B2 at 5 and 10 μg/ml each for a duration of 24 h when compared to the untreated control cells demonstrating no neurotoxic effect of antibody treatment alone. All scatter bar graphs are mean ± SEM.

Supplementary Figure 2 DC take up tau antibodies through receptor-mediated endocytosis. NDC and DC were co-incubated with 300 μg/mL 10,000 MW Alexa 568 tagged Dextran and 20 μg/mL Alexa 488 tagged 6B2 tau antibody for 24 h. Cells were then washed, incubated with Hoechst stain to visualize nuclei, washed, and fixed with paraformaldehyde for microscopy. **(A,B)** Shows confocal microscopy images of NDC and DC, respectively. The merge panel exemplifies the pronounced co-localization of dextran and tau antibody in NDC, while there was significantly less co-localization in DC. Both sets of images were analyzed for intensity correlation coefficient between the dextran and 6B2 signals. The colocalization analyses revealed strong colocalization in NDC as seen by the merged image, and strong intensity correlation between 6B2 and dextran (*R*^2^ = 0.729), while limited colocalization was seen in DC as reflected by a weak intensity correlation coefficient (*R*^2^ = 0.123). As indicated in the differentiated merged blow-up image, the green dots are much more prominent than the orange/yellow dots that reflect colocalization. Also, since the differentiated cells take up much less dextran because of their limited bulk-endocytosis, the red dots are smaller and not well discerned from the yellow/orange dots to the naked eye. In parallel, 6B2 tau antibody (5 μg/mL) and increasing doses of a clathrin-mediated endocytosis inhibitor, Dynasore (1–5 μg/mL) or vehicle (DMSO) were co-incubated with NDC or DC for 24 h. Cells were then lysed and collected for Western blot analyses. **(C,D)** Shows representative Western blots of NDC and DC treated with Dynasore and probed for anti-mouse IgG1 and GAPDH. **(E,F)** Shows the quantified results of the Western blots where the anti-mouse IgG1 signal was normalized to GAPDH. In NDC, Dynasore did not significantly affect antibody uptake at any dose, whereas it did in DC at the 5 μg/mL dosage (39% decrease, ^*^*p* = 0.04, *n* = 7–8, *t*-test, one-tailed) compared to the vehicle control. All scatter bar graphs are mean ± SEM.

Supplementary Figure 3 PHF is dose-dependently taken up by NDC. NDC were treated with 0–10 μg/mL Alexa Fluor 647 tagged PHF-tau for 24 h. Cells were then collected and examined with flow cytometry. **(A)** Cells were gated for viable and singlet cells. **(B)** Furthermore, cells were analyzed for Alexa Fluor 647 positive cells, generating histograms for each sample, % of PHF positive cells and Median Fluorescence Intensity (MFI). **(C)** Shows dose-dependent increase in percent PHF positive cells, which plateaued between 1 and 10 μg/mL. **(D)** Quantification of MFI of the Alexa Fluor 647-tagged PHF, normalized to the 0 μg/mL dosage, which shows a dose-dependent increase in the intracellular PHF signal. All plots are mean ± SEM.

Supplementary Video 1 Time-lapse live imaging shows time-dependent colocalization of PHF and 4E6 in pre-treated DC. DC were pre-incubated with 50 μg/mL tagged PHF-AF488 for 16 h, prepared for live imaging as described above, and subsequently treated with 20 μg/mL Cypher5E (a pH sensitive dye)-tagged 4E6 tau antibody for up to 140 min. Time dependent internalization of 4E6 occurred primarily in the soma, and progressively co-occupied areas where PHF resided in the soma since the beginning of the experiment. Colocalization increased over time, as indicated by the yellow color.

Supplementary Video 2 Time-lapse live imaging shows 6B2 uptake in pre-treated DC, but it does not colocalize with PHF. DC were pre-incubated with 50 μg/mL tagged PHF-AF488 for 16 h, prepared for live imaging as described above, and subsequently treated with 20 μg/mL Cypher5E (a pH sensitive dye)-tagged 6B2 tau antibody for up to 150 min. Time-dependent internalization of 6B2 occurred primarily in the neurites, while PHF resided in the soma since the beginning of the experiment.

Supplementary Video 3 Time-lapse live imaging shows IgG1 uptake in pre-treated DC, but it does not colocalize with PHF. DC were pre-incubated with 50 μg/mL tagged PHF-AF488 for 16 h, prepared for live imaging as described above, and subsequently treated with 20 μg/mL Cypher5E (a pH sensitive dye)-tagged IgG1 control antibody for up to 150 min. Time-dependent internalization of IgG1 occurred primarily in the neurites, while PHF resided in the soma since the beginning of the experiment.

## References

[1] Congdon EE Sigurdsson EM. Tau-targeting therapies for Alzheimer disease. Nat Rev Neurol. (2018) 14:399–415. 10.1038/s41582-018-0013-z 29895964 PMC6463489

[2] Bittar A Bhatt N Kayed R. Advances and considerations in AD tau-targeted immunotherapy. Neurobiol Dis. (2020) 134:104707. 10.1016/j.nbd.2019.104707 31841678 PMC6980703

[3] Colin M Dujardin S Schraen-Maschke S Meno-Tetang G Duyckaerts C Courade JP. From the prion-like propagation hypothesis to therapeutic strategies of anti-tau immunotherapy. Acta Neuropathol. (2020) 139:3–25. 10.1007/s00401-019-02087-9 31686182 PMC6942016

[4] Sandusky-Beltran LA Sigurdsson EM. Tau immunotherapies: Lessons learned, current status and future considerations. Neuropharmacology. (2020) 175:108104. 10.1016/j.neuropharm.2020.108104 32360477 PMC7492435

[5] Asuni AA Boutajangout A Quartermain D Sigurdsson EM. Immunotherapy targeting pathological tau conformers in a tangle mouse model reduces brain pathology with associated functional improvements. J Neurosci. (2007) 27:9115–29. 10.1523/JNEUROSCI.2361-07.2007 17715348 PMC6672191

[6] Boutajangout A Ingadottir J Davies P Sigurdsson EM. Passive immunization targeting pathological phospho-tau protein in a mouse model reduces functional decline and clears tau aggregates from the brain. J Neurochem. (2011) 118:658–67. 10.1111/j.1471-4159.2011.07337.x 21644996 PMC3366469

[7] Gu J Congdon EE Sigurdsson EM. Two novel Tau antibodies targeting the 396/404 region are primarily taken up by neurons and reduce Tau protein pathology. J Biol Chem. (2013) 288:33081–95. 10.1074/jbc.M113.494922 24089520 PMC3829157

[8] Funk KE Mirbaha H Jiang H Holtzman DM Diamond MI. Distinct therapeutic mechanisms of Tau antibodies: promoting microglial clearance vs. blocking neuronal uptake. J Biol Chem. (2015) 290:21652–62. 10.1074/jbc.M115.657924 26126828 PMC4571888

[9] Luo W Liu W Hu X Hanna M Caravaca A Paul SM. Microglial internalization and degradation of pathological tau is enhanced by an anti-tau monoclonal antibody. Sci Rep. (2015) 5:11161. 10.1038/srep11161 26057852 PMC4460904

[10] Andersson CR Falsig J Stavenhagen JB Christensen S Kartberg F Rosenqvist N. Antibody-mediated clearance of tau in primary mouse microglial cultures requires Fcγ-receptor binding and functional lysosomes. Sci Rep. (2019) 9:4658. 10.1038/s41598-019-41105-4 30874605 PMC6420568

[11] Holmes BB Diamond MI. Prion-like properties of Tau protein: the importance of extracellular Tau as a therapeutic target. J Biol Chem. (2014) 289:19855–61. 10.1074/jbc.R114.549295 24860099 PMC4106306

[12] Congdon E Lin Y Rajamohamedsait H Shamir D Rajamohamedsait W Rasool S. Affinity of tau antibodies for soluble pathological tau species but not their immunogen or insoluble tau aggregates predicts *in vivo* and *ex vivo* efficacy. Mol Neurodegener. (2016) 30:62. 10.1186/s13024-016-0126-z 27578006 PMC5006503

[13] Krishnamurthy PK Deng Y Sigurdsson EM. Mechanistic studies of antibody-mediated clearance of tau aggregates using an *ex vivo* brain slice model. Front Psychiatry. (2011) 2:59. 10.3389/fpsyt.2011.00059 22025915 PMC3198029

[14] Congdon EE Gu J Sait HB Sigurdsson EM. Antibody uptake into neurons occurs primarily via clathrin-dependent Fcgamma receptor endocytosis and is a prerequisite for acute tau protein clearance. J Biol Chem. (2013) 288:35452–65. 10.1074/jbc.M113.491001 24163366 PMC3853292

[15] Collin L Bohrmann B Gopfert U Oroszlan-Szovik K Ozmen L Gruninger F. Neuronal uptake of tau/pS422 antibody and reduced progression of tau pathology in a mouse model of Alzheimer's disease. Brain. (2014) 137(Pt 10):2834–46. 10.1093/brain/awu213 25085375

[16] Krishnaswamy S Lin Y Rajamohamedsait WJ Rajamohamedsait HB Krishnamurthy P Sigurdsson EM. Antibody-derived *in vivo* imaging of tau pathology. J Neurosci. (2014) 34:16835–50. 10.1523/JNEUROSCI.2755-14.2014 25505335 PMC4261104

[17] Kondo A Shahpasand K Mannix R Qiu J Moncaster J Chen C-H. Antibody against early driver of neurodegeneration cis P-tau blocks brain injury and tauopathy. Nature. (2015) 523:431–6. 10.1038/nature14658 26176913 PMC4718588

[18] Shamir DB Deng Y Sigurdsson EM Dynamics of intracellular interaction of a tau antibody and human pathological tau in a human neuron-like model [Abstract]. In: Society for Neuroscience Annual Meeting Abstract Number 199.10. San Diego, CA (2016).

[19] Shamir DB Rosenqvist N Rasool S Pedersen JT Sigurdsson EM. Internalization of tau antibody and pathological tau protein detected with a flow cytometry multiplexing approach. Alzheimer's Dementia. (2016) 12:1098–107. 10.1016/j.jalz.2016.01.013 27016263 PMC5383206

[20] McEwan WA Falcon B Vaysburd M Clift D Oblak AL Ghetti B. Cytosolic Fc receptor TRIM21 inhibits seeded tau aggregation. Proc Natl Acad Sci USA. (2017) 114:574–9. 10.1073/pnas.1607215114 28049840 PMC5255578

[21] Nisbet RM Van der Jeugd A Leinenga G Evans HT Janowicz PW Götz J. Combined effects of scanning ultrasound and a tau-specific single chain antibody in a tau transgenic mouse model. Brain. (2017) 140:1220–30. 10.1093/brain/awx052 28379300 PMC5405237

[22] Wu Q Lin Y Gu J Sigurdsson EM. Dynamic assessment of tau immunotherapies in the brains of live animals by two-photon imaging. EBioMedicine. (2018) 35:270–8. 10.1016/j.ebiom.2018.08.041 30146345 PMC6158769

[23] Congdon EE Chukwu JE Shamir DB Deng J Ujla D Sait HBR. Tau antibody chimerization alters its charge and binding, thereby reducing its cellular uptake and efficacy. EBioMedicine. (2019) 42:157–73. 10.1016/j.ebiom.2019.03.033 30910484 PMC6492224

[24] Krishnaswamy S Huang HW Marchal IS Ryoo HD Sigurdsson EM. Neuronally expressed anti-tau scFv prevents tauopathy-induced phenotypes in Drosophila models. Neurobiol Dis. (2020) 137:104770. 10.1016/j.nbd.2020.104770 31982516 PMC7178494

[25] Lee VMY Wang J Trojanowski JQ [6] Purification of paired helical filament tau and normal tau from human brain tissue. In: Wetzel R editor. Methods in Enzymology. Cambridge, MA: Academic Press (1999). p. 81–9. 10.1016/s0076-6879(99)09008-4 10507018

[26] Rostagno A. Ghiso J. (2009). Isolation and biochemical characterization of amyloid plaques and paired helical filaments. Curr Protoc Cell Biol. Chapter 3, Unit 3.33 3.33.1-33. 10.1002/0471143030.cb0333s44 PMC2793596 19731227

[27] Chukwu JE Congdon EE Sigurdsson EM Kong XP. Structural characterization of monoclonal antibodies targeting C-terminal Ser(404) region of phosphorylated tau protein. MAbs. (2019) 11:477–88. 10.1080/19420862.2019.1574530 30794086 PMC6512906

[28] Encinas M Iglesias M Liu Y Wang H Muhaisen A Ceña V. Sequential treatment of SH-SY5Y cells with retinoic acid and brain-derived neurotrophic factor gives rise to fully differentiated, neurotrophic factor-dependent, human neuron-like cells. J Neurochem. (2000) 75:991–1003. 10.1046/j.1471-4159.2000.0750991.x 10936180

[29] Jamsa A Hasslund K Cowburn RF Backstrom A Vasange M. The retinoic acid and brain-derived neurotrophic factor differentiated SH-SY5Y cell line as a model for Alzheimer's disease-like tau phosphorylation. Biochem Biophys Res Commun. (2004) 319:993–1000. 10.1016/j.bbrc.2004.05.075 15184080

[30] Agholme L Lindstrom T Kagedal K Marcusson J Hallbeck M. An *in vitro* model for neuroscience: differentiation of SH-SY5Y cells into cells with morphological and biochemical characteristics of mature neurons. J Alzheimers Dis. (2010) 20:1069–82. 10.3233/JAD-2010-091363 20413890

[31] Shamir DB Deng Y Sigurdsson EM. Live imaging of pathological tau protein and tau antibodies in a neuron-like cellular model. Methods Mol Biol. (2018) 1779:371–9. 10.1007/978-1-4939-7816-8_22 29886544

[32] Li Q Lau A Morris TJ Guo L Fordyce CB Stanley EF. A syntaxin 1, Galpha(o), and N-type calcium channel complex at a presynaptic nerve terminal: analysis by quantitative immunocolocalization. J Neurosci. (2004) 24:4070–81. 10.1523/JNEUROSCI.0346-04.2004 15102922 PMC6729428

[33] Adler J Parmryd I. Quantifying colocalization by correlation: the Pearson correlation coefficient is superior to the Mander's overlap coefficient. Cytometry A. (2010) 77:733–42. 10.1002/cyto.a.20896 20653013

[34] Krishnaswamy S Wu Q Lin Y Rajamohamedsait WJ Rajamohamedsait HB Sigurdsson EM. *In vivo* imaging of tauopathy in mice. Methods Mol Biol. (2018) 1779:513–26. 10.1007/978-1-4939-7816-8_32 29886554

[35] Karuppagounder SS Shi Q Xu H Gibson GE. Changes in inflammatory processes associated with selective vulnerability following mild impairment of oxidative metabolism. Neurobiol Dis. (2007) 26:353–62. 10.1016/j.nbd.2007.01.011 17398105 PMC2753424

[36] Modak SR Solesio M Krishnaswamy S Congdon EE Sigurdsson EM Antibodies targeting truncated tau protein reduce tau pathology in primary neuronal and mixed cortical cultures. In: Society for Neuroscience Annual Meeting #579.14. Chicago, IL (2015).

[37] Shamir DB Sigurdsson E Tau antibodies reduce tau levels in differentiated but not in non-differentiated human SH-SY5Y cells. In: Society for Neuroscience Abstract 579.15. Chicago, IL (2015).

[38] Lesuisse C Martin LJ. Long-term culture of mouse cortical neurons as a model for neuronal development, aging, and death. J Neurobiol. (2002) 51:9–23. 10.1002/neu.10037 11920724

[39] Park S Jung Y. Combined actions of Na/K-ATPase, NCX1 and glutamate dependent NMDA receptors in ischemic rat brain penumbra. Anat Cell Biol. (2010) 43:201–10. 10.5115/acb.2010.43.3.201 21212860 PMC3015038

[40] McKiernan RC Jimenez-Mateos EM Bray I Engel T Brennan GP Sano T. Reduced mature microRNA levels in association with dicer loss in human temporal lobe epilepsy with hippocampal sclerosis. PLoS ONE. (2012) 7:e35921. 10.1371/journal.pone.0035921 22615744 PMC3352899

[41] Murphy KE Gysbers AM Abbott SK Tayebi N Kim WS Sidransky E. Reduced glucocerebrosidase is associated with increased alpha-synuclein in sporadic Parkinson's disease. Brain. (2014) 137(Pt 3):834–48. 10.1093/brain/awt367 24477431 PMC3927701

[42] Ryu HW Park CW Ryu KY. Disruption of polyubiquitin gene Ubb causes dysregulation of neural stem cell differentiation with premature gliogenesis. Sci Rep. (2014) 4:7026. 10.1038/srep07026 25391618 PMC4229674

[43] Bruhns P Jönsson F. Mouse and human FcR effector functions. Immunol Rev. (2015) 268:25–51. 10.1111/imr.12350 26497511

[44] Gustafsson G Eriksson F Möller C da Fonseca TL Outeiro TF Lannfelt L. Cellular uptake of α-synuclein oligomer-selective antibodies is enhanced by the extracellular presence of α-synuclein and mediated via Fcγ receptors. Cell Mol Neurobiol. (2017) 37:121–31. 10.1007/s10571-016-0352-5 26961542 PMC11482100

[45] Proctor DT Coulson EJ Dodd PR. Reduction in post-synaptic scaffolding PSD-95 and SAP-102 protein levels in the Alzheimer inferior temporal cortex is correlated with disease pathology. J Alzheimers Dis. (2010) 21:795–811. 10.3233/JAD-2010-100090 20634587

[46] Shao CY Mirra SS Sait HB Sacktor TC Sigurdsson EM. Postsynaptic degeneration as revealed by PSD-95 reduction occurs after advanced Abeta and tau pathology in transgenic mouse models of Alzheimer's disease. Acta Neuropathol. (2011) 122:285–92. 10.1007/s00401-011-0843-x 21630115 PMC3437675

[47] Yuki D Sugiura Y Zaima N Akatsu H Takei S Yao I. DHA-PC and PSD-95 decrease after loss of synaptophysin and before neuronal loss in patients with Alzheimer's disease. Sci Rep. (2014) 4:7130. 10.1038/srep07130 25410733 PMC5382699

[48] Ji Y Pang PT Feng L Lu B. Cyclic AMP controls BDNF-induced TrkB phosphorylation and dendritic spine formation in mature hippocampal neurons. Nat Neurosci. (2005) 8:164–72. 10.1038/nn1381 15665879

[49] Nagappan G Lu B. Activity-dependent modulation of the BDNF receptor TrkB: mechanisms and implications. Trends Neurosci. (2005) 28:464–71. 10.1016/j.tins.2005.07.003 16040136

[50] Yoshii A Constantine-Paton M. Postsynaptic BDNF-TrkB signaling in synapse maturation, plasticity, and disease. Dev Neurobiol. (2010) 70:304–22. 10.1002/dneu.20765 20186705 PMC2923204

[51] Cao C Rioult-Pedotti MS Migani P Yu CJ Tiwari R Parang K. Impairment of TrkB-PSD-95 signaling in Angelman syndrome. PLoS Biol. (2013) 11:e1001478. 10.1371/annotation/f32bc670-c9cf-4bb0-9376-cd8cfd1053c1 23424281 PMC3570550

[52] Yoshii A Constantine-Paton M. BDNF induces transport of PSD-95 to dendrites through PI3K-AKT signaling after NMDA receptor activation. Nat Neurosci. (2007) 10:702–11. 10.1038/nn1903 17515902

[53] Yoshii A Murata Y Kim J Zhang C Shokat KM Constantine-Paton M. TrkB and protein kinase Mzeta regulate synaptic localization of PSD-95 in developing cortex. J Neurosci. (2011) 31:11894–904. 10.1523/JNEUROSCI.2190-11.2011 21849550 PMC3158490

[54] Laske C Stransky E Leyhe T Eschweiler GW Maetzler W Wittorf A. BDNF serum and CSF concentrations in Alzheimer's disease, normal pressure hydrocephalus and healthy controls. J Psychiatr Res. (2007) 41:387–94. 10.1016/j.jpsychires.2006.01.014 16554070

[55] Santa-Maria I Varghese M Ksiezak-Reding H Dzhun A Wang J Pasinetti GM. Paired helical filaments from Alzheimer disease brain induce intracellular accumulation of Tau protein in aggresomes. J Biol Chem. (2012) 287:20522–33. 10.1074/jbc.M111.323279 22496370 PMC3370237

[56] Wu JW Herman M Liu L Simoes S Acker CM Figueroa H. Small misfolded Tau species are internalized via bulk endocytosis and anterogradely and retrogradely transported in neurons. J Biol Chem. (2013) 288:1856–70. 10.1074/jbc.M112.394528 23188818 PMC3548495

[57] Michel CH Kumar S Pinotsi D Tunnacliffe A St George-Hyslop P Mandelkow E. Extracellular monomeric tau protein is sufficient to initiate the spread of tau protein pathology. J Biol Chem. (2014) 289:956–67. 10.1074/jbc.M113.515445 24235150 PMC3887218

