# Supplementary figures and images for: Dynamics of Internalization and Intracellular Interaction of Tau Antibodies and Human Pathological Tau Protein in a Human Neuron-Like Model
Source: Front Neurol. 2020 Nov 26;11:602292. doi: 10.3389/fneur.2020.602292 (PMC7727311; doi:10.3389/fneur.2020.602292)

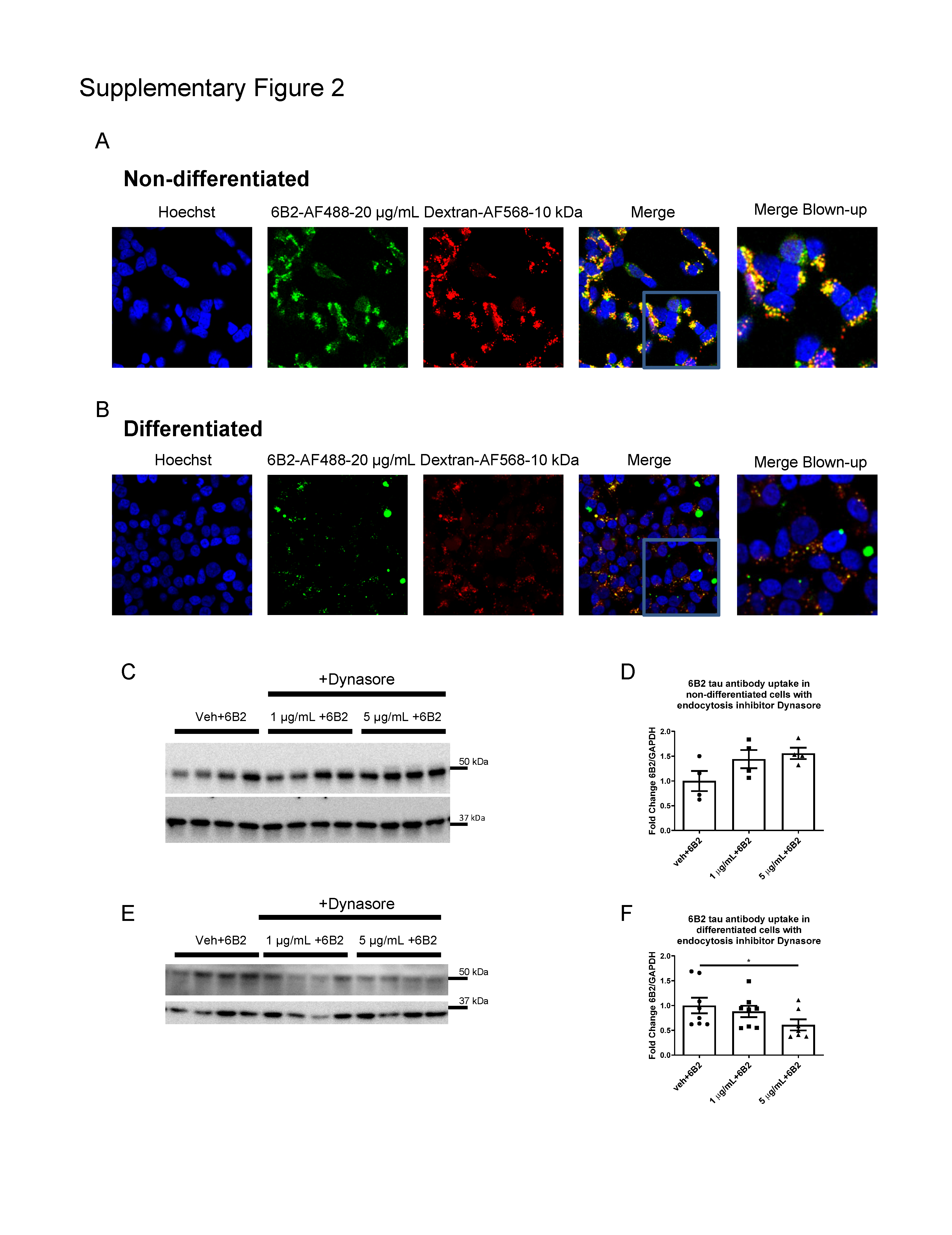

Supplement: Supplementary Figure 2 — DC take up tau antibodies through receptor-mediated endocytosis. NDC and DC were co-incubated with 300 μg/mL 10,000 MW Alexa 568 tagged Dextran and 20 μg/mL Alexa 488 tagged 6B2 tau antibody for 24 h. Cells were then washed, incubated with Hoechst stain to visualize nuclei, washed, and fixed with paraformaldehyde for microscopy. (A,B) Shows confocal microscopy images of NDC and DC, respectively. The merge panel exemplifies the pronounced co-localization of dextran and tau antibody in NDC, while there was significantly less co-localization in DC. Both sets of images were analyzed for intensity correlation coefficient between the dextran and 6B2 signals. The colocalization analyses revealed strong colocalization in NDC as seen by the merged image, and strong intensity correlation between 6B2 and dextran (R2 = 0.729), while limited colocalization was seen in DC as reflected by a weak intensity correlation coefficient (R2 = 0.123). As indicated in the differentiated merged blow-up image, the green dots are much more prominent than the orange/yellow dots that reflect colocalization. Also, since the differentiated cells take up much less dextran because of their limited bulk-endocytosis, the red dots are smaller and not well discerned from the yellow/orange dots to the naked eye. In parallel, 6B2 tau antibody (5 μg/mL) and increasing doses of a clathrin-mediated endocytosis inhibitor, Dynasore (1–5 μg/mL) or vehicle (DMSO) were co-incubated with NDC or DC for 24 h. Cells were then lysed and collected for Western blot analyses. (C,D) Shows representative Western blots of NDC and DC treated with Dynasore and probed for anti-mouse IgG1 and GAPDH. (E,F) Shows the quantified results of the Western blots where the anti-mouse IgG1 signal was normalized to GAPDH. In NDC, Dynasore did not significantly affect antibody uptake at any dose, whereas it did in DC at the 5 μg/mL dosage (39% decrease, *p = 0.04, n = 7–8, t-test, one-tailed) compared to the vehicle control. All scatter ba [file Image_2.tiff]
